# Investigation of Human Intrathecal Solute Transport Dynamics Using a Novel *in vitro* Cerebrospinal Fluid System Analog

**DOI:** 10.3389/fnimg.2022.879098

**Published:** 2022-06-23

**Authors:** Akari Seiner, Goutham Kumar Reddy Burla, Dev Shrestha, Mayumi Bowen, Joshua D. Horvath, Bryn A. Martin

**Affiliations:** ^1^Department of Chemical and Biological Engineering, University of Idaho, Moscow, ID, United States; ^2^Genentech, Inc., A Member of the Roche Group, South San Francisco, CA, United States; ^3^Alcyone Therapeutics Inc., Lowell, MA, United States

**Keywords:** cerebrospinal fluid—CSF, intrathecal (i.t.) injection, central nervous system, *in vitro*, drug delivery & targeting, lumbar puncture (LP), biofluid mechanics, neuroimaging (anatomic)

## Abstract

**Background:**

Understanding the relationship between cerebrospinal fluid (CSF) dynamics and intrathecal drug delivery (ITDD) injection parameters is essential to improve treatment of central nervous system (CNS) disorders.

**Methods:**

An anatomically detailed *in vitro* model of the complete CSF system was constructed. Patient-specific cardiac- and respiratory-induced CSF oscillations were input to the model in the subarachnoid space and within the ventricles. CSF production was input at the lateral ventricles and CSF absorption at the superior sagittal sinus. A model small molecule simulated drug product containing fluorescein was imaged within the system over a period of 3-h post-lumbar ITDD injections and used to quantify the impact of (a) bolus injection volume and rate, (b) post-injection flush volume, rate, and timing, (c) injection location, and (d) type of injection device. For each experiment, neuraxial distribution of fluorescein in terms of spatial temporal concentration, area-under-the-curve (AUC), and percent of injected dose (%ID) to the brain was quantified at a time point 3-h post-injection.

**Results:**

For all experiments conducted with ITDD administration in the lumbar spine, %ID to the brain did not exceed 11.6% at a time point 3-h post-injection. Addition of a 12 mL flush slightly increased solute transport to the brain up to +3.9%ID compared to without a flush (*p* < 0.01). Implantation of a lumbar catheter with the tip at an equivalent location to the lumbar placed needle, but with rostral tip orientation, resulted in a small improvement of 1.5%ID to the brain (*p* < 0.05). An increase of bolus volume from 5 to 20 mL improved solute transport to the brain from 5.0 to 6.3%ID, but this improvement was not statistically significant. Increasing bolus injection rate from 5 to 13.3 mL/min lacked improvement of solute transport to the brain, with a value of 6.3 compared to 5.7%ID.

**Conclusion:**

The *in vitro* modeling approach allowed precisely controlled and repeatable parametric investigation of ITDD injection protocols and devices. In combination, the results predict that parametric changes in lumbar spine ITDD-injection related parameters and devices can alter %ID to the brain and be tuned to optimize therapeutic benefit to CNS targets.

## Background

According to the World Health Organization, disorders of the CNS including neuroinflammatory, neurodegenerative, and neurovascular conditions impact ~1 billion people in the world, making it the world's leading cause of disability (Soderquist and Mahoney, 2010; Calias et al., 2014; Khani et al., 2020b). Many of these neurological disorders require treatment. However, the human body's blood-brain barrier (BBB) tightly regulates transport of substances from the blood to the brain to precisely control CNS homeostasis (Daneman and Prat, 2015), often rendering oral and parenteral drug administration ineffective (Calias et al., 2014; Pizzichelli et al., 2017). To overcome the BBB, two prominent strategies have been proposed: (1) the development of drugs that can pass through the barrier; and (2) utilizing alternative drug delivery routes, such as intracerebroventricular, intranasal, intra-cisterna magna, and intrathecal drug delivery (ITDD) (Calias et al., 2014).

### Intrathecal Drug Delivery

At present, ITDD is primarily utilized clinically for the treatment of three conditions: (1) chronic non-malignant pain; (2) muscle spasticity; and (3) cancer related pain (Shah and Padalia, 2019). Subcutaneous ports, electronic pumps or single dose injections through the interspinous ligaments in the L3–L5 region are common administration methods for ITDD (Belov et al., 2021). ITDD has been considered a viable option in solving the brain drug delivery problem as it allows for bypassing of the BBB to directly access the CNS (Brumback, 1988; Verma et al., 2020). It works by delivering the drug directly into the cerebrospinal fluid (CSF) within the spinal subarachnoid space (SAS). This route has been found to potentially reduce the required dose 100–300X compared to oral administration, which may lead to lower drug toxicity and reduced side effects while retaining a therapeutic dose to targets of interest within the CNS (Simpson and Jones, 2008; Soderquist and Mahoney, 2010; Calias et al., 2014; Gulur et al., 2014). Furthermore, ITDD is often considered less invasive compared to other drug delivery methods such as intracerebroventricular and intraparenchymal administrations (Belov et al., 2021) and has shown to potentially be more effective (Calias et al., 2012).

ITDD has been investigated in many research studies. A study by Whiteside et al. investigated the efficacy of hyperbaric solutions as spinal anesthesia *via* the intrathecal route (Whiteside et al., 2001). Previous studies have shown that lumbar intrathecal (IT) administration into the CSF reached the hypothalamus in baboons and dogs (LeBel et al., 1999; McCarthy et al., 2002). A study by Munoz-Rojas et al. found that a patient with Hunter syndrome who received IT injections of loranidase showed improved walk test distance and pulmonary parameters, decreased numbing and tingling, increased stability, and a decreased need for pain medication (Munoz-Rojas et al., 2008). Another study by Muenzer et al. showed that, after 6 months, mean CSF glycosaminoglycans reduced in patients with Hunter syndrome after intrathecal treatment of idursulfase-IT (Muenzer et al., 2016). The use of ITDD for cancer patients has also been investigated (Smith et al., 2002; Rauck et al., 2003). The benefits associated with ITDD compared to conventional pain medication administration methods allowed patients to undergo more aggressive chemotherapy/radiation treatment and henceforth increased patient life expectancy (Smith et al., 2002; Deer et al., 2011). The Food and Drug Administration approved morphine, ziconotide, and baclofen for use *via* the intrathecal route (Bottros and Christo, 2014). In 2016, nusinersen became the first approved drug to treat spinal muscular atrophy, and it is administered intrathecally as a 5 mL dose within the lumbar SAS (Claborn et al., 2019; Neil and Bisaccia, 2019; Li, 2020). Currently, there are several clinical trials that are investigating intrathecal chemotherapy (Qian, 2016a,b, 2021), stem cell therapy (Kurtzberg, 2014; Staff, 2017; Rong, 2018a,b,c; Lu, 2020; Prodromos, 2021), gene therapy (Sehgal, 2021), and others (McCarthy and Charlesworth, 2017; NIH, 2021).

### Modeling of CSF Transport

A thorough understanding of CSF flow dynamics may lead to improved detection and treatment of CNS disorders (Khani et al., 2019, 2020b). Solute transport in the CSF has been shown to play an important role in ensuring drug distribution to the target site (Jose et al., 2013). CSF is a clear fluid that resides in the SAS of the brain and spine (Martin and Heidari Pahlavian, 2019). It has several important purposes, including acting as a “shock absorber” to stabilize intracranial pressure (Yildiz et al., 2017; Martin and Heidari Pahlavian, 2019), providing protection and suspension of neural tissue (Khani et al., 2019), and maintaining immunological and biochemical homeostasis (Engelhardt and Coisne, 2011). The importance of CSF dynamics in CNS disorders has been investigated in several studies (Hårdemark et al., 1988; Klimo et al., 2004; Freedman et al., 2005; Hatterer et al., 2008; Zetterberg et al., 2013; Simon and Iliff, 2016). It is believed that CSF is produced primarily within the ventricles and absorbed at the arachnoid granulations on the surface of the superior sagittal sinus (del Bigio, 2010). CSF pulsates in sync with intracranial cardiac and respiratory cycles and has approximately zero net flow (Martin and Heidari Pahlavian, 2019; Khani et al., 2020b).

Several modeling studies have been conducted to assess intrathecal solute transport in the spinal CSF. In 1996, Myers used an idealized 3D elliptical geometry to investigate the impact of injection flow rate, catheter size, and catheter angle (Myers, 1996). An early study by Tangen et al. (2015) used a patient specific SAS model to investigate the effects of spinal microanatomy on flow patterns and stirring effects. A later study by Tangen et al. (2017) investigated the effect of injection volume and CSF pulsations. Hsu et al. (2012) assessed the impacts of CSF pulsations on ITDD using a 2D geometric model from anatomical images. Haga et al. (2017) and Pizzichelli et al. (2017) investigated the effect of catheter position and angle, tissue permeability, and injection flow rates. Kuttler et al. investigated the impact of a slow vs. fast bolus (Kuttler et al., 2010).

A precise understanding of the impact of ITDD injection parameters on pharmacokinetics may help further optimize intrathecal solute transport and provide drug distribution to the target site of pharmacologic action (Hocking and Wildsmith, 2004; Kuttler et al., 2010; Tangen et al., 2017). Therefore, the goal of the present study was to investigate the impact of several lumbar puncture (LP)-based injection parameters and the injection device types on intrathecal solute transport to the brain in a subject-specific 3D anatomically detailed human *in vitro* model of the SAS. To our knowledge, this is the first study to investigate a combination of ITDD infusion parameters that include injection volumes and rates, injection location, and injection device. The results of this study provide potential guidelines for intrathecal administration of drugs and further optimize ITDD injection protocols to improve solute transport to the brain for the treatment of CNS disorders.

## Methods

The overarching approach was to utilize a subject-specific 3D human model to investigate the impact of the following parameters on solute distribution to the brain *via* CSF using a simulated small molecule model of fluorescein: (a) bolus injection volume and rate, (b) flush volume, rate, and timing, (c) injection location, and (d) type of device (Table 1). For the purpose of this study, the brain was considered the target region for therapeutic benefit of the injected solute. Each experiment was conducted over the course of 3-h and the solute distribution was observed *via* a spatial temporal slice average concentration and quantified in terms of the percent of the injected dose (%ID). A period of 3-h was chosen to potentially represent initial pharmacokinetic transport of the solute within the CSF, neglecting biology of solute uptake into the CNS tissue. A small molecule simulated drug product was utilized in this study; depending on the type of injected molecule, the biological half-life could be variable.

**Table 1:** List of protocols to test the injection parameters.

**Exp.** **num**.	**Exp.** **name**	**Inj.** **loc**.	**Inj.** **conc.** **(μM)**	**Inj.** **dir**.	**Device**	**Bolus** **vol.** **(mL)**	**Bolus** **rate** **(mL/min)**	**Flush** **vol.** **(mL)**	**Flush** **rate** **(mL/min)**
1	1HUM1	L3–L4	331.02	PA	LP	5	5	0	0
2	1HUM2	L3–L4	331.02	PA	LP	5	5	*5*	2.5
3	1HUM3	L3–L4	331.02	PA	LP	5	5	5	*5*
4	2HUM1	L3–L4	82.75	PA	LP	20	5	0	0
5	2HUM2	L3–L4	82.75	PA	LP	20	*13.3*	0	0
6	3HUM1	L3–L4	331.02	IS	*Cath*	5	5	0	0
7	3HUM2	L3–L4	82.75	IS	Cath	20	7	2	2.5
8	3HUM3	L3–L4	331.02	IS	Cath	5	5	*12*	5
9	4HUM2	*L1–L2*	331.02	IS	Cath	5	5	12	5
10	5HUM1	L3–L4	331.02	PA	LP	5	5	*15*	5
11	5HUM4	L3–L4	331.02	PA	LP	5	5	*15 mL, 15 min*	5

*Italics indicates the change in parameter between protocols. PA represents the posterior-anterior direction, IS represents the inferior to superior direction. LP represents lumbar puncture, indicating the needle was utilized*.

### Model Geometry

The development of the subject-specific 3D model used in this study has been previously described by Sass et al. (2017) and Khani et al. (2020b). In brief, a healthy 23-year-old female underwent a high-resolution T2 weighted magnetic resonance (MR) imaging sequence that was used to quantify subject-specific CSF space geometry 5656. The model was developed by combining the result of the high-resolution MR images with 31 pairs of anatomically realistic dorsal and ventral nerve roots, thecal sac, and filum terminale (Sass et al., 2017; Khani et al., 2020b). Stereolithography was used to print the model as a 2 mm thick transparent shell in three parts: cranial, upper thoracic, and lower spine, to avoid exceeding the limit of the 3D printer (Khani et al., 2020b). Once combined, the model was 76 cm in length and had a total CSF space volume of ~330 mL. A detailed description of the subject-specific model, including the connectivity of cervical and lumbar nerve roots, the ventricular system (lateral, third, and fourth ventricles), cerebellum, basal cistern, and the cortical SAS can be found in Khani et al. (2022).

### Overall System Layout

**Figure 1:**
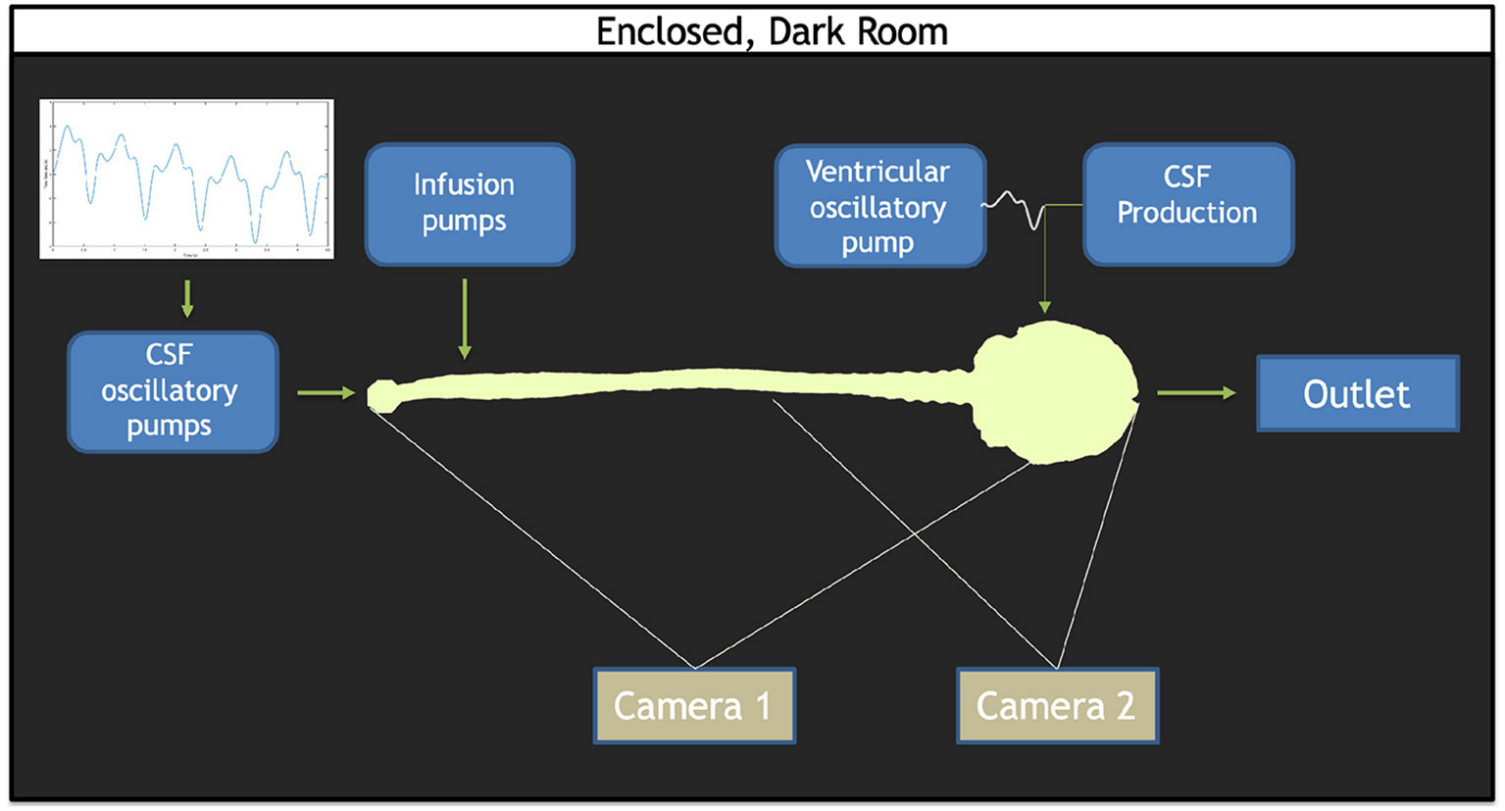
Overview of the *in vitro* model set up. The CSF waveform comprised of the cardiac and respiratory components is imposed by the CSF oscillatory pumps. Infusion pumps consisting of both the bolus and flush pumps are located at the lumbar region. A ventricular oscillatory pump utilizes the identical CSF oscillations at the caudal end, but at 1/36th the scale. A CSF production pump set to infuse 0.4 mL/min into the ventricles. A CSF reservoir tank to capture the overflow rests at the cranial end of the model. Two cameras were used: one to capture the full span of the model (Camera 1) and one to capture the lower concentrations within the brain (Camera 2).

The *in vitro* system layout previously used by our group has been briefly described by Khani et al. (2020b, 2022). In brief, the following system modifications were made in this study: (a) the addition of two infusion pumps for implementation of the bolus injection and flush, (b) CSF waveform verification, (c) utilization of two cameras to image the system, and (d) implementation of an algorithm for image stitching from the two cameras. A block diagram of the *in vitro* system configuration is shown in Figure 1.

#### Flow Input Boundary Conditions and Verification

Cardiac- and respiratory-induced CSF flow oscillations have been found to lead to solute dispersion along the neuroaxis (Kuttler et al., 2010). Therefore, a respiration component of CSF pulsation was combined with the cardiac components of CSF pulsations under natural breathing for this study through a pulsatile waveform derived from Yildiz et al. (2017). A custom computer-controlled pump was used to input cardiac- and respiratory-induced CSF oscillations within the SAS. A separate pump was used to input cardiac- and respiratory-induced CSF oscillations within the ventricles of the brain (Sass et al., 2017; Khani et al., 2018, 2020b). An additional off-the-shelf pump continuously infused 0.4 mL/min representing CSF production in the lateral ventricles, a CSF production rate previously quantified in the literature (Figure 1). Two off-the-shelf syringe pumps were used to administer the simulated drug product bolus and flush, when utilized (Huang et al., 2004; Brinker et al., 2014; Liu et al., 2020). To mimic solute transport in the CSF, an aqueous solution of fluorescein sodium was injected to represent a small molecule drug product. The usage of fluorescein as an injection tracer has been applied in previous studies (Sun et al., 2002; Bagger and Bechgaard, 2004; Aaron and Trajkovska, 2006). Given that solute transport within the CSF is dependent on pulsations and vorticity, thereby independent of the chemical composition of the bulk fluid, all experiments and solute injections were conducted using deionized water as the working fluid (Tangen et al., 2016).

To quantify CSF oscillatory waveform reliability, five repetitions using an inline flow sensor (Transonic, 4PXN) with a multi-channel research console (Transonic, T402) was conducted. Similarly, a smaller inline flow sensor (Transonic, 1PXN) was used to conduct five repetitions to verify CSF waveform imparted to the ventricles. All infusion and CSF production pumps were verified *via* a stopwatch and bucket test before and after all experiments were conducted.

### Imaging Configuration, Calibration, and Post-processing

**Figure 2:**
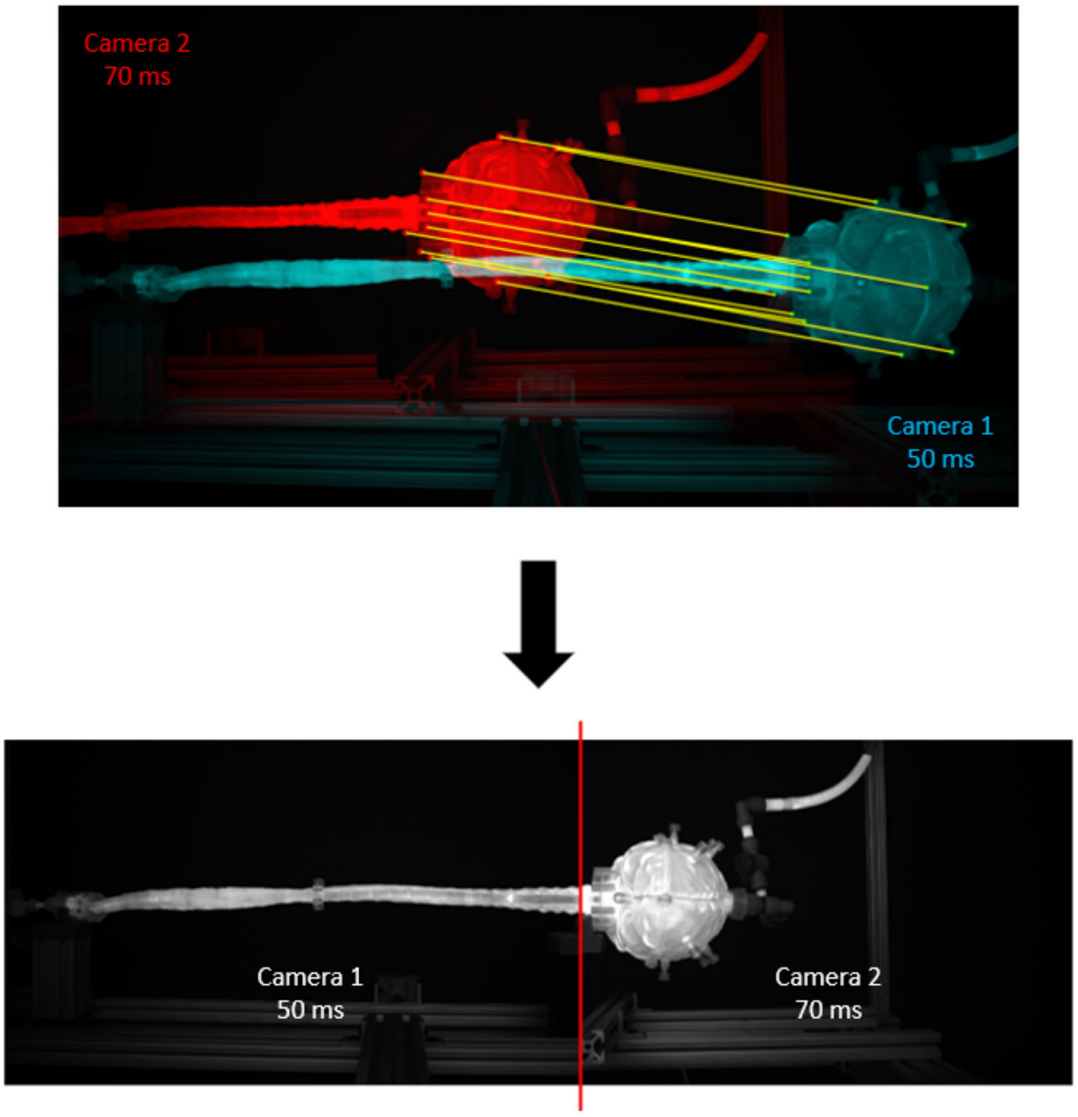
Results of the image stitching process. Twelve matching points from each camera are manually segmented and input to a MATLAB stitching algorithm to produce a stitched image at the C1 level. Camera 1 corresponds to the full model camera; camera 2 corresponds to the brain camera. There are twelve matching points, including: (1) brain cap center, (2) top port, (3) bottom port, (4) top of the cap, (5) bottom of the cap, (6) phalange middle, (7) phalange top, (8) flange bottom, (9) left mount corner, (10) very top of the flange, (11) very bottom of the flange, (12) right mount corner.

Imaging configuration, calibration, and post-processing was applied as previously described by Khani et al. (2020a,b). In brief, an imaging camera was used to quantify axial distribution of fluorescein tracer concentration over time. The imaging system utilized two high-resolution cameras with one focused on the brain and the other focused on the spine (Figure 1). Each camera was optimized to attain maximum dynamic range within its respective imaging locations by modification of exposure time. The capturing of images was triggered by cardiac systole to obtain consistent phase acquisition during each CSF flow oscillation. A mask was applied to each camera image to define the model edges for the brain and spine. A manually draw ROI was then specified at the craniocervical junction to delimit the brain only and spine only. To calibrate the system, the model was filled with known molar concentrations of fluorescein spanning the range of dilution within the *in vitro* model and imaged so that the raw pixel intensities could be converted to molar concentrations. The collected images were used to interpolate pixel intensity to molar concentrations for each location and time. To analyze both images from the brain and spine camera, the images were stitched (Figure 2).

### Outline of the Injection Parameters

The parameters investigated included (a) bolus injection rate and volume, (b) flush volume, rate, and timing, (c) infusion device as either LP needle or lumbar catheter, and (d) injection location within the lumbar spine (Table 2). The baseline bolus injection volume and rate were set to 5 and 5 mL/min, respectively (1HUM1), a value based on the protocol administered for nusinersen in spinal muscular atrophy (Neil and Bisaccia, 2019). The 1HUM1 condition was utilized as a standard for which to make comparisons across experiments.

**Table 2:** Experimental design of tested ITDD injection parameters.

**Num**.	**Parameter**	**Protocol 1**	**Protocol 2**	**Range** **analyzed**
1	Bolus volume	1HUM1	2HUM1	5–20 mL
2	Bolus rate	2HUM1	2HUM2	5–13.3 mL/min
3	Flush volume	3HUM1	3HUM3	0–12 mL
4	Flush rate	1HUM2	1HUM3	2.5–5 mL/min
5	Device	1HUM1	3HUM1	Needle–Cath
6	Injection location	3HUM3	4HUM2	L3/L4–L1/L2
7	Flush timing	5HUM1	5HUM4	0–15 min

*The parameter, its associated protocols, and the range analyzed are included*.

To study the effect of bolus injection volume, the bolus injection rate was held constant at 5 mL/min and the bolus volume increased by four times from 5 to 20 mL (1HUM1–2HUM1). Injection bolus rate was observed by increasing the rate from 5 to 13.3 mL/min while maintaining a constant 20 mL bolus volume (2HUM1–2HUM2). To study the effect of flush volume, a 12 mL flush was added to the baseline case with the flush administered subsequent to the initial bolus injection (3HUM1–3HUM3). The impact of flush rate was investigated by increasing the rate from 2.5 to 5 mL/min (1HUM2–1HUM3). The effect of injection device type was observed by switching the 3.5″ 22-gauge spinal needle (~0.4 mm ID, Jorgensen Labs, J-529 H, SNM1018-046) in the baseline case to a 3.5 Fr (0.6 mm ID × 1.1 mm OD) rounded tip polyurethane catheter (Access Technologies, CNC-3.5PR-36″) (1HUM1–3HUM1). Injection location was compared by moving the catheter tip two vertebral levels from L3–L4 to L1–L2 (3HUM3–4HUM2). The impact of a delayed flush was also compared by injecting a 15 mL flush immediately after the bolus injection and subsequent to a 15-min delay following the bolus injection (5HUM1–5HUM4).

### Repeatability and Reliability

To verify repeatability of experimental results three repetitions for each experiment were conducted with an average time delay of 6 days between each repetition. A detailed comparison of repetitions was performed by the following correlation analysis: the standard deviation of the slice average concentration for all three repetitions at each z-location and time was calculated and plotted as a spatial temporal plot to visualize the location of variability between repetitions. Additionally, an array of the slice average concentration mean at each z-location and time for all three repetitions was generated and each individual repetition of an experiment was subtracted from this mean and plotted as a Bland-Altman plot in terms of spatial temporal differences of concentration and location over 3-h post-injection. The standard deviation and the 95% confidence interval (CI) of the mean of the repetitions was calculated. Furthermore, the error as a percent of dynamic range (% DR) was calculated as:


(1) $$\begin{array}{r} \%\;DR=\;\frac{95\%\;CI}{C_{\max}}*100 \end{array}$$

where C_max_ represents the maximum concentration. Ideally, the % DR is <5%.

### Quantification of Intrathecal Solute Transport

To quantify the intrathecal solute transport to the brain at 3-h post-injection, the percent injected dose to the brain (%ID) was calculated as:


(2) $$\begin{array}{r} Injection\;Mass\;\left(g\right)=Injection\;Volume\;\left(L\right)\;*\;Injection\;Concentration\;\left(\frac{g}{L}\right) \end{array}$$

(3) $$\begin{array}{r} Slice\;Mass\;\left(g\right)=Slice\;Volume\;\left(L\right)\;*\;Slice\;Concentration\;\left(\frac{g}{L}\right) \end{array}$$

(4) $$\begin{array}{r} Slice\;Sum\;\left(g\right)=\Sigma \;Slice\;Mass\;\left(g\right) \end{array}$$

(5) $$\begin{array}{r} \%ID\;to\;brain\;=\left(\frac{Slice\;Sum}{Total\;Injected\;Mass}\right)\;*\;100 \end{array}$$

The brain portion of the model was defined as the anatomic region superior to the craniocervical junction located at model position defined as 0 cm. Solute exposure to the brain was calculated as the trapezoidal integral value of the tracer concentration over 3-h and is hereon referred to as the area under the curve (AUC).

### Statistical Analysis

The mean, standard deviation, and 95% CI in terms of %ID was calculated for each experiment. To assess the significance of a given parameter, the pooled variance was calculated using the following analysis. First, a variance check was conducted in STATGRAPHICS, and the variances were found to be equal. The common standard deviation was then estimated by the pooled standard deviation. The populations were assumed to be independent and normal, such to allow the assumption that the population followed a t-distribution with n_1_ + n_2_-2 degrees of freedom. The (1–a) 100% confidence interval for m_1_-m_2_ for pooled variances could be calculated as: $\bar{x_{1}}-\bar{x_{2}}\;\pm \;t_{\frac{\alpha }{2}\;}s_{p}\sqrt{\frac{1}{n_{1}}+\;\frac{1}{n_{2}}}\;$, where x_1_ is the mean from sample 1, x_2_ is the mean from sample 2, $t_{\frac{\alpha }{2}}$ comes from a t-distribution with n_1_ + n_2_ – 2 degrees of freedom, s_p_ is the pooled standard deviation, and n_1_ and n_2_ correspond to the sample sizes of population 1 and 2, respectively. A one-way analysis of variance in MATLAB was conducted to test significant differences between parameters. An a = 0.05 was used to indicate significance.

## Results

### Verification of Boundary Conditions and Repeatability

**Figure 3:**
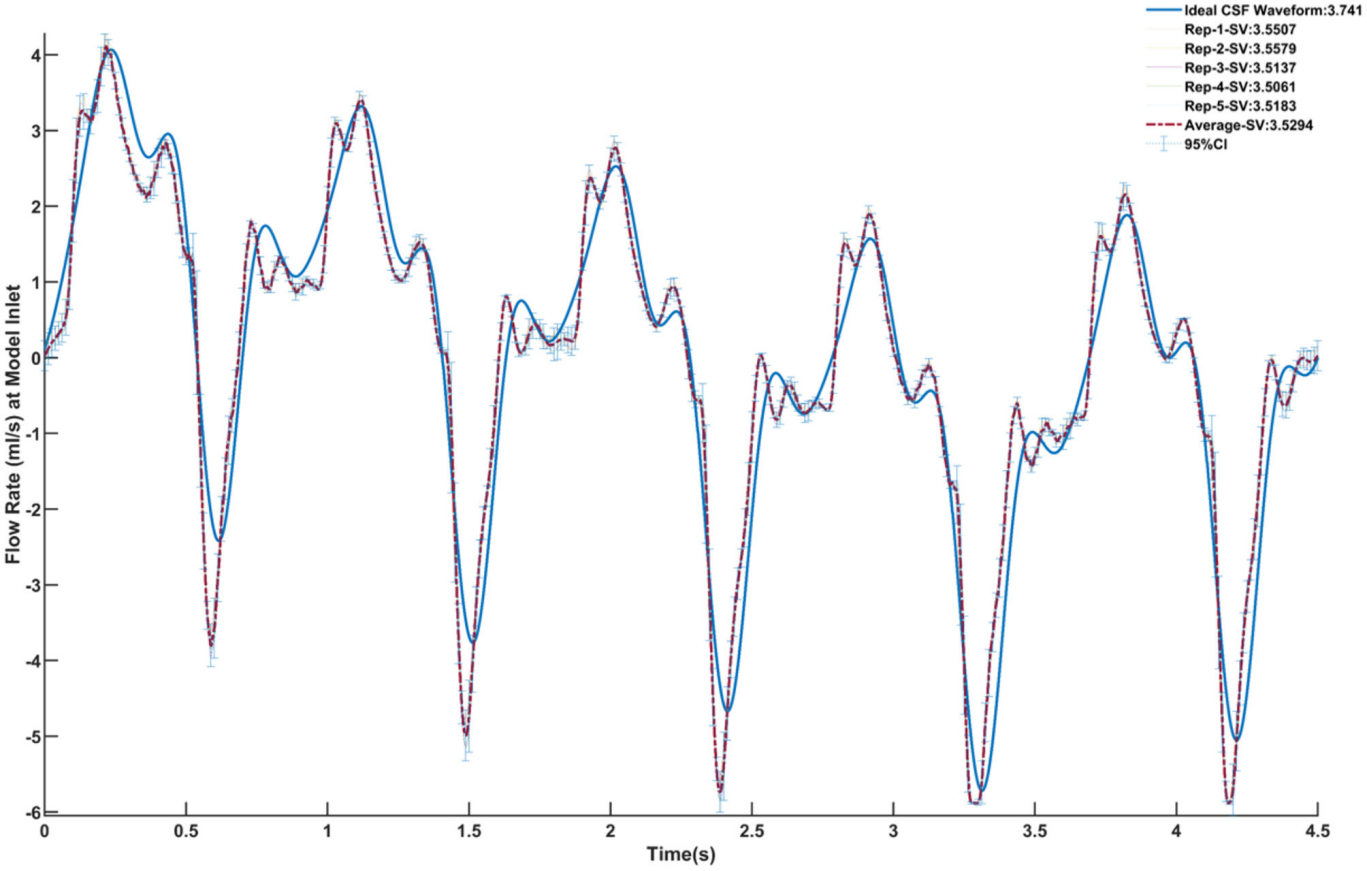
Verification of the CSF oscillatory waveform at the caudal end of the model using five repetitions. The ideal waveform derived from MRI measurements is outlined in blue. Each repetition is shown, and the relative stroke volume is calculated. The average of all five repetitions is outlined in the red dashed line. The 95% confidence interval for the average of the repetitions is also shown.

**Figure 4:**
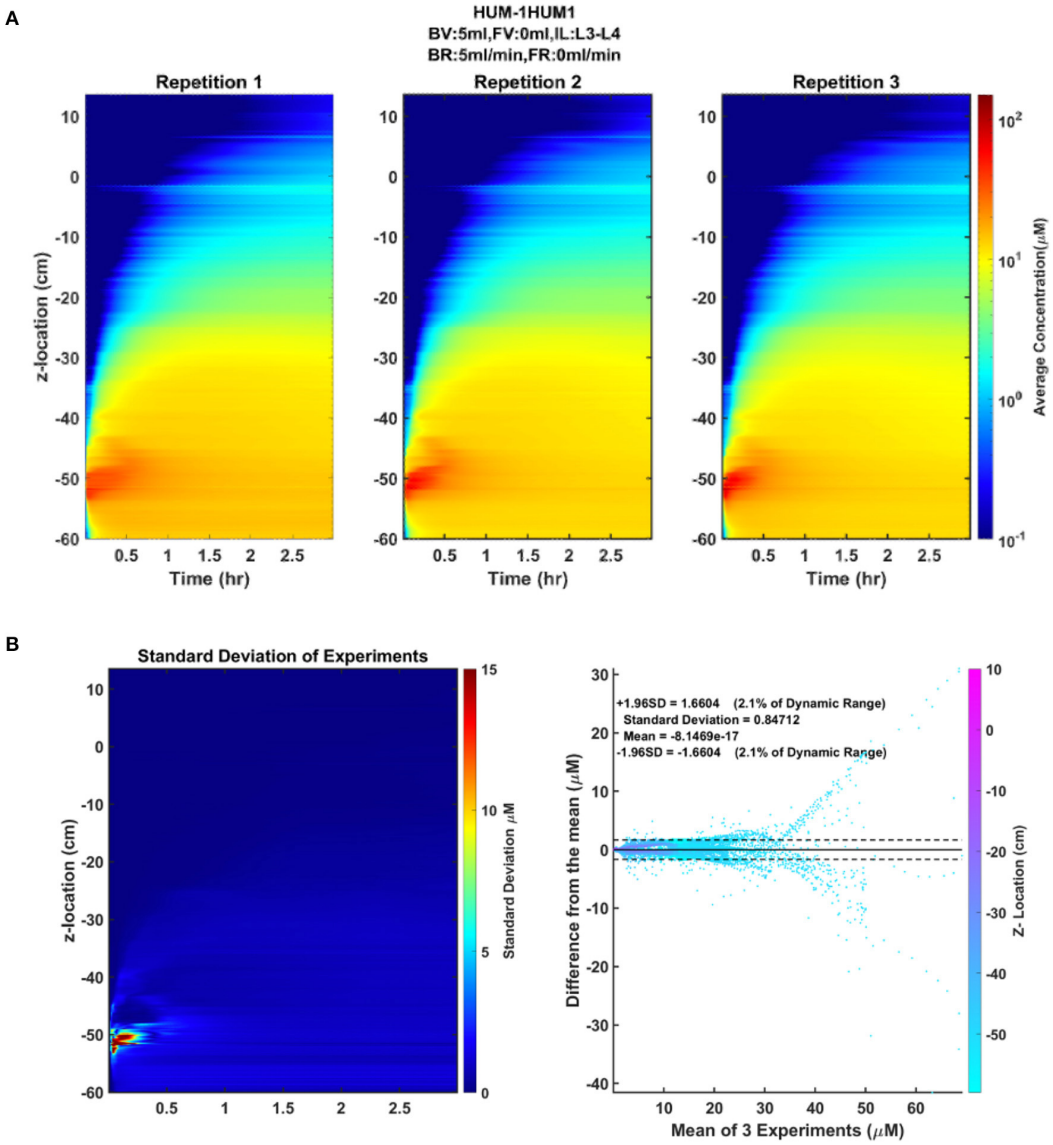
Repeatability and reliability across repetitions of an experiment. **(A)** The spatial temporal plot representing the average concentration of each slice by position and time for each repetition is shown to visualize the distribution of the solute over time. **(B)** The standard deviation spatial temporal plot indicating the regions of greatest variance across the three repetitions, in addition to a Bland-Altman plot showing the difference of each repetition from the mean of the repetitions.

The CSF oscillatory waveforms were verified using the Transonic flow meter with agreement between the input of the *in vitro* model and the ideal waveform (Figure 3). A stopwatch and bucket test was used to calculate the error as percent of average CSF production rate and was found to be <0.02%. Strong repeatability of the slice average concentration was seen between experiment repetitions (Table 3). The greatest standard deviation and 95% CI observed was 1.68 and 3.29 micromoles, respectively, and error as a %DR did not exceed 6.87% for all experiments conducted (Table 3). Agreement of solute distribution with respect to time and location across repetitions can be observed as a spatial temporal plot in Figure 4A. The location with greatest variance for repeated experiments was near the injection location (Figure 4B).

**Table 3:** Repeatability and reliability of the slice average concentration between the repetitions for each *in vitro* experiment.

**Exp. num**.	**Exp. name**	**By (μM)**		**By %ID**	
		**|SD| (μM)**	**95% CI (μM)**	**|SD| (%ID)**	**95% CI (%ID)**
			**(Error as %DR)**		
1	1HUM1	0.7965	1.561 (2.03%)	0.65	1.28
2	1HUM2	1.1319	2.218 (1.82%)	0.69	1.36
3	1HUM3	0.6484	1.271 (1.01%)	0.55	1.08
4	2HUM1	1.6804	3.294 (3.15%)	1.00	1.96
5	2HUM2	1.6301	3.195 (2.23%)	0.41	0.80
6	3HUM1	1.2837	2.516 (5.2%)	0.40	0.78
7	3HUM2	0.6595	1.293 (2.4%)	1.26	2.47
8	3HUM3	0.7450	1.460 (3.01%)	1.35	2.64
9	4HUM2	0.9928	1.946 (1.24%)	1.42	2.79
10	5HUM1	1.476	2.893 (6.87%)	1.18	2.32
11	5HUM4	1.3723	2.689 (4.26%)	2.46	4.83

*The standard deviation and 95% confidence interval are calculated in terms of micromoles and %ID*.

### Effect of Injection Parameters

**Figure 5:**
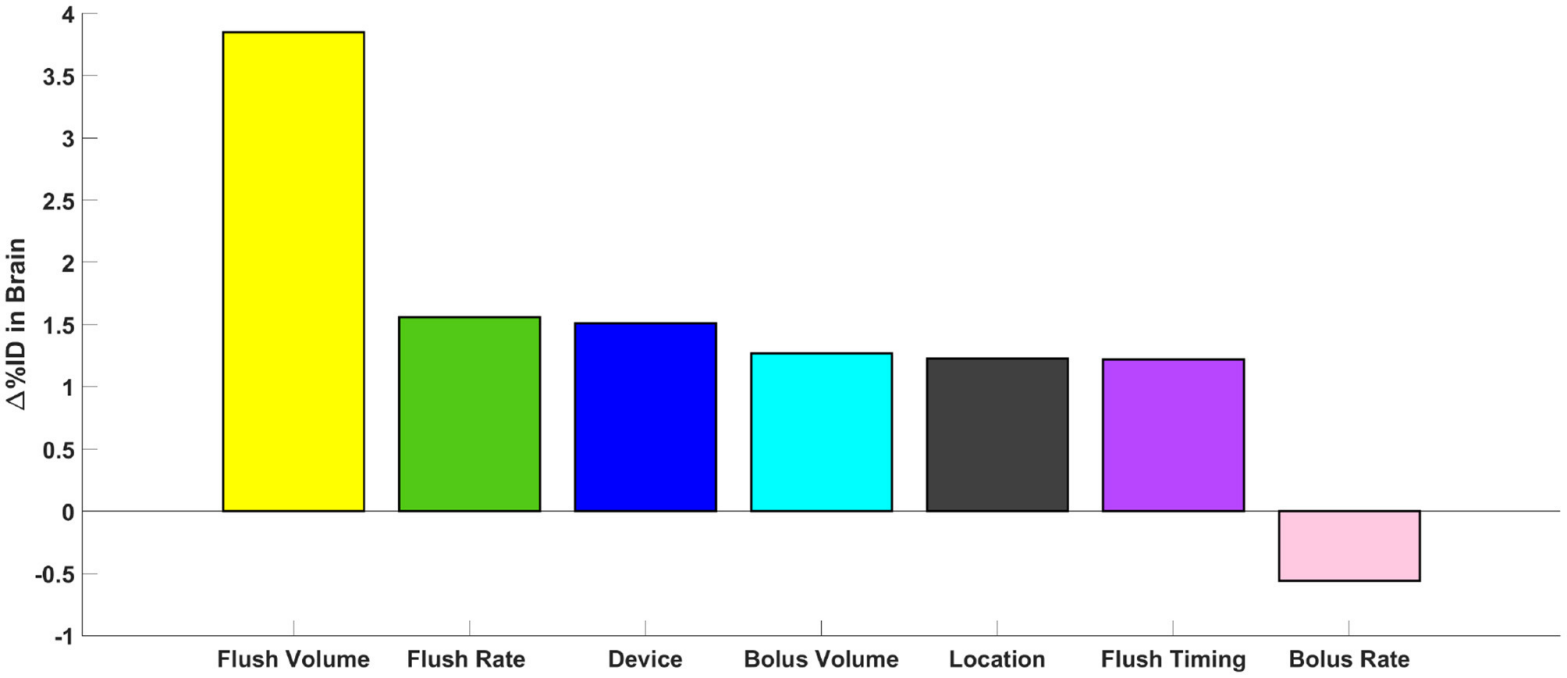
Parametric comparison of parameters. Represented as the change in %ID in the brain at 3-h, the impact of all parameters tested can be visualized.

Impact of injection parameters on solute transport to the brain are shown in Table 4. From the conducted protocols, flush volume was the most important factor leading to increased solute delivery to the brain, followed by flush rate, bolus injection volume, location, device, and bolus injection rate (Figure 5). By increasing the flush volume from 0 to 12 mL (3HUM1–3HUM3), solute transport increased to the brain by 3.9% (95% CI 1.6–6.1%ID, *p* = 0.009). Additional flush volume protocols were conducted, and similar trends were observed. An increase of flush rate from 2.5 to 5 mL/min (1HUM2–1HUM3) increased 1.6%ID (95% CI 0.2–2.9%ID, *p* = 0.038). Using a catheter increased solute transport to the brain by 1.5%ID (95% CI 0.3–2.7%ID, *p* = 0.026). By increasing the bolus injection volume from 5 to 20 mL (2HUM1–2HUM2) increased solute transport to the brain by 1.3% (95% CI −0.7–3.2%ID). Moving the catheter tip from L3–L4 to L1–L2 (3HUM3–4HUM2) increased by 1.2% (95% CI−1.9–4.4%ID). An additional location experiment was conducted, and similar trends were observed. An increase in bolus injection rate had the least impact on solute delivery to the brain; an increase from 5 to 13.3 mL/min (2HUM1–2HUM2) showed a decrease in %ID to the brain by 0.6% (95% CI −2.3–1.2%ID). Similar results were observed when comparing the average AUC in the brain at 3-h (Table 4). An increase of 1.2%ID (95% CI −3.2–5.6%ID) was seen with a delayed flush. The bolus injection volume, type of device, location of injection, and bolus injection rate were not statistically significant. In addition, %ID observed in the protocols relating to a lumbar catheter (3HUM1 without flush-−4HUM2 with flush) ranged from 6.0 to 11.6%, while the %ID observed in the protocols relating to a LP needle (1HUM1 with lower bolus injection volume and rate-−2HUM2 with higher bolus injection volume and rate) ranged 4.9–6.5% (*p* < 0.0001). Supplementary Figures 1–8 show the average spatial temporal tracer concentration and AUC trends for each experiment described in Table 1.

**Table 4:** Quantification of parametric comparison by ranking.

**Rank**	**Parameter**	**Range of parameter analyzed**	**Protocol names**	**%ID to brain @ 3-h** **(Δ%ID)**	**Avg brain AUC** **(μM-h) @ 3-h** **(ΔAUC)**	**Statistical significance,** **α = 0.05**
1	Flush volume	0 vs. 12 mL	3HUM1 vs. 3HUM3	6.53 vs. 10.38 (+3.85)	0.67 vs. 1.20 (+0.53)	*p* = 0.009*
2	Flush rate	2.5 vs. 5 mL/min	1HUM2 vs. 1HUM3	4.90 vs. 6.46 (+1.56)	0.50 vs. 0.64 (+0.14)	*p* = 0.038*
3	Device	Needle vs. Cath	1HUM1 vs. 3HUM1	5.02 vs. 6.53 (+1.51)	0.48 vs. 0.67 (+0.19)	*p* = 0.026*
4	Bolus volume	5 vs. 20 mL	1HUM1 vs. 2HUM1	5.02 vs. 6.29 (+1.27)	0.48 vs. 0.66 (+0.18)	*p* = 0.14
5	Location	L3/L4 vs. L1/L2	3HUM3 vs. 4HUM2	11.61 vs. 10.38 (+1.23)	1.20 vs. 1.37 (+0.17)	*p* = 0.39
6	Flush timing	0 vs. 15 min	5HUM1 vs. 5HUM4	6.00 vs. 7.22 (+1.22)	0.69 vs. 0.86 (+0.17)	*p* = 0.48
7	Bolus rate	5 vs. 13.3 mL/min	2HUM1 vs. 2HUM2	6.29 vs. 5.73 (−0.56)	0.66 vs. 0.65 (−0.01)	*p* = 0.42

Relevant information includes the parameter investigated, range of the given parameter, the names of the protocols used to investigate the parameter, and the %ID and average AUC in the brain at 3-h for each protocol. The change in %ID and average AUC are calculated and used to compare the impact of the given parameter. *Denotes statistical significance.

### AUC Trends and Impact of Time Duration Analyzed

**Figure 6:**
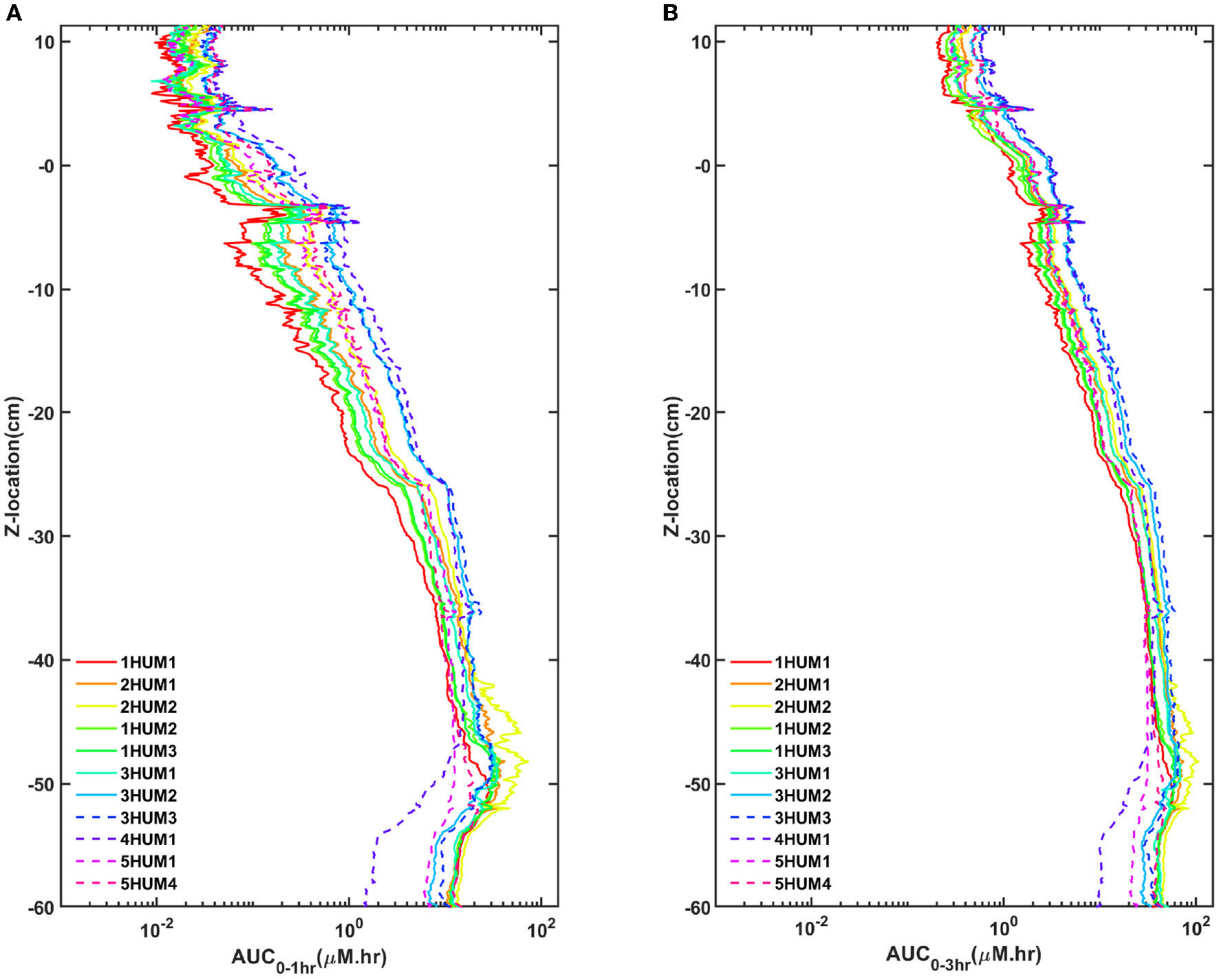
AUC trends for all cases. **(A)** One-hour and 3-h for all cases and **(B)** 1- and 3-h for the best/worst case. There is good agreement of the trends and a significant difference between the best and worst case.

**Figure 7:**
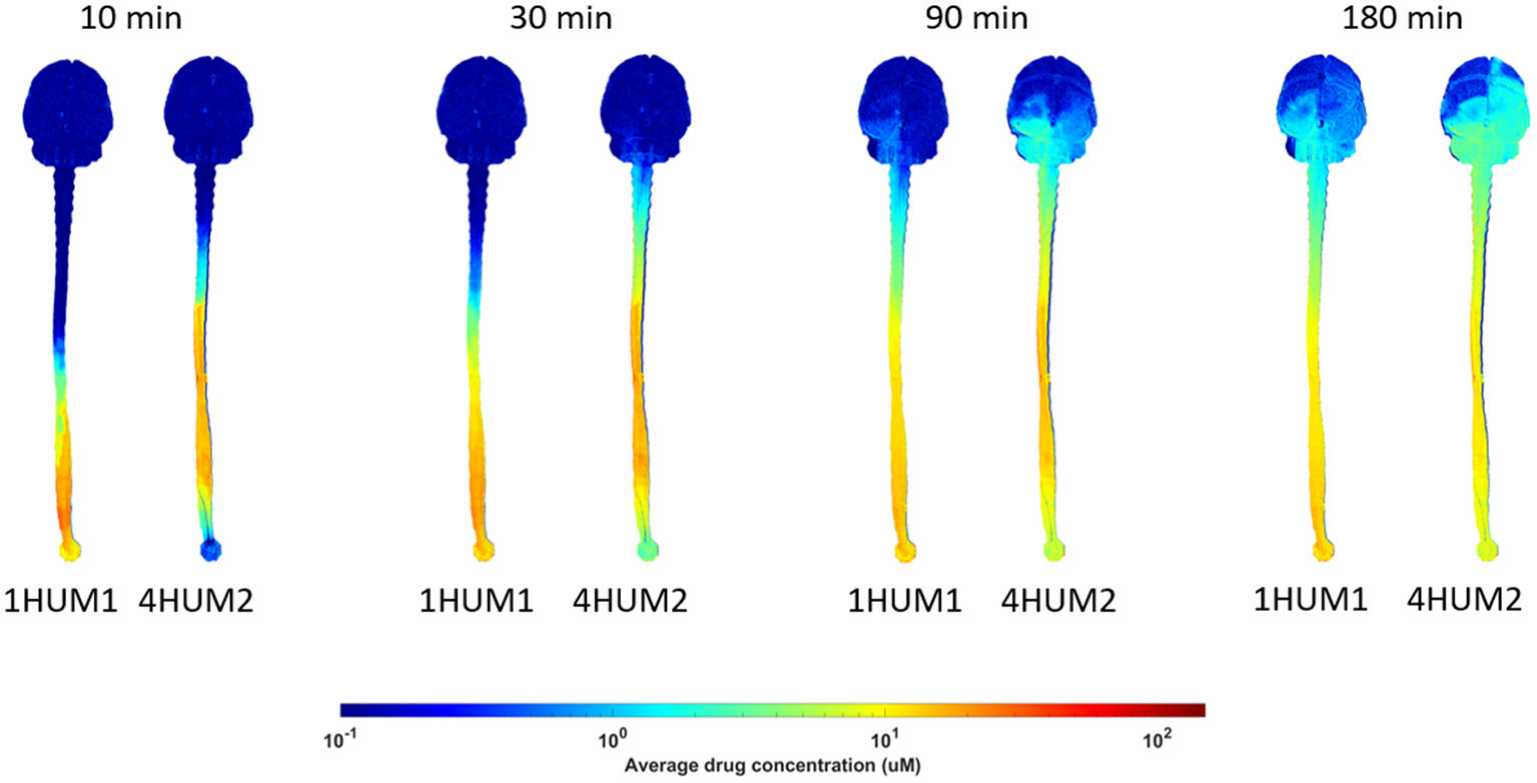
Difference in distribution over time. Solute transport for 1HUM1 (left) and 4HUM2 (right) are shown at 10, 30 min, 1.5-, and 3-h. There is a significant difference between the protocols at the 10 and 30 min time points, however, at 1.5- and 3-h, this difference decreases.

Across all experiments, a ~2 log difference in regional AUC was present between the spine compared to the brain at a time point 3-h after injection (Figure 6). AUC values near the injection site were greatest for all experiments and AUC values around the brain dropped precipitously with a minimum amount located near the superior aspect of the brain. Protocols with the greatest solute transport to the brain displayed the lower AUC values in the lumbar region at 1- and 3-h post-injection (Figure 6). For all cases, the solute distribution differences decreased over time (Figure 7). In 1HUM1 at 10-min, the solute spread to approximately the thoracic region (T8/T9); in 4HUM2 at 10-min, the solute started to spread to the cervical region (C7). However, at 3-h post-injection, the distribution of the solute appeared similar with both cases spreading around the brain, to some degree.

## Discussion

Intrathecal drug delivery to the brain has been increasingly utilized and investigated in part due to its potential for comparably low required dosages to achieve the desired pharmacological response (Shah and Padalia, 2019). This may potentially translate into increased efficacy and reduced side effects relative to oral and parenteral administration of the same agent (Tangen et al., 2017; Shah and Padalia, 2019). Despite this trend, there still exists a dearth of knowledge regarding the effect of injection parameters on solute transport. Therefore, guidelines to assist clinicians in the selection of specific administration parameters may further improve treatment outcomes for patients (Tangen et al., 2017). Our approach was to model early drug pharmacokinetic distribution within the CSF, neglecting drug absorption into the tissue of the CNS, using a subject-specific human patient model to parametrically assess the effects of (1) bolus injection volume and rate; (2) flush volume, rate, and timing; (3) lumbar spine injection location; and (4) type of device. The key findings of this study include:

1. Under all injection scenarios analyzed, relatively little solute reached the brain (<12%ID).
2. Flush volume increased solute distribution to the brain to a greater degree than other injection parameters analyzed.
3. Increasing bolus injection rate did not increase %ID solute to the brain.
4. Use of a lumbar placed catheter for injection of the solute resulted in greater solute distribution to the brain compared to a lumbar puncture needle with identical tip location along the spine, but different orientation.

All parameters analyzed showed some impact on solute transport to the brain; however, the impact for any individual parameter was relatively small (the greatest increase did not exceed 3.9% ID). The case that showed the greatest solute transport to the brain was 4HUM2, which utilized a combination of injection parameters that each improved %ID to the brain including: (a) an injection location two vertebral levels closer to the brain, (b) a lumbar placed catheter with tip oriented toward the brain, and (c) an increased flush volume. Thus, combination of factors could potentially be used to further optimize solute transport to the brain for lumbar ITDD.

### Parametric Investigation of ITDD Delivery Parameters Showed Poor Brain Drug Delivery Efficiency

Across all experiments conducted in this study, the goal was to maximize the amount of solute transport to the brain. Regardless of protocol, all LP simulations resulted in limited distribution to the brain. Indeed, the range of %ID observed in the brain at 3-h post-injection was 5.0–11.6% across the protocols tested. Even with several parametric delivery injections analyzed, this indicates a relatively poor brain delivery efficiency of lumbar ITDD, with most of the injected solute remaining in the lumbar spine 3-h post-injection. In the case of spinal cord injury or disease, this may be a desirable result as the target is within the spinal region. In cases of targeting the brain, the efficiency of solute transport observed in this study was, on average, 7.2%ID and dosing decisions would need to account for this limited distribution. Parametric changes in %ID induced by flush volume, rate, and timing, bolus volume and rate, location of lumbar injection, and type of lumbar device did modify solute transport to the brain by −0.6–3.9%ID. While these changes are small, the degree of change in terms of percent from standard LP injection protocol is notable. Indeed, if 5% of the total solute is delivered to the brain, a parametric change in %ID of 3.9% equates to a 78% increase in solute transport to the brain. In this context, one can see how readily optimization of solute transport to the brain can be attained. This demonstrates a potential in which a relatively modest change in injection parameter(s) can make substantial percentwise improvements in brain delivery.

### Flush Volume Was the Single Most Important Factor for Solute Transport of Lumbar Injections

Flushing the injection device may help rinse out residual drug volume and ensure device integrity and is therefore often part of ITDD protocols (Hemley et al., 2012; Chung et al., 2016; Slavc et al., 2018). Our results showed that an increase in flush volume, within the lumbar spine after initial bolus injection, significantly (*p* = 0.009) increased solute distribution to the brain (Figure 5). Tangen et al. utilized a computational model to investigate two protocols related to flush (Tangen et al., 2017). Their first protocol, with a 5 mL bolus injection and a 5 mL flush, showed high drug concentration spread between the C3 and T5 region; their second protocol, with a 5 mL bolus injection and a 10 mL flush at the same injection rate, showed spread to the upper cervical spine and the brain parenchyma, improving drug delivery to the brain within 1-h post-injection (Tangen et al., 2017). Similar protocols were conducted for our *in vitro* study relating to flush volume. For both protocols conducted in our study with the 5 mL flush, the solute spread reached the C1–C4 levels at 1-h post-injection, and for a higher flush volume of 15 mL, the solute reached the cranial base within 1-h post-injection; a value in agreement with the Tangen study.

The potential benefit of a lumbar spine flush has been documented in several pre-clinical studies (Hinderer et al., 2014). A study by Wolf et al. utilized a variety of imaging methods to track neuraxial exposure following an IT lumbar bolus injection in rats (Wolf et al., 2016). A protocol consisting of a 30 μL bolus followed by a 40 μL saline flush was tested. Immediately following injection, the bolus filled the spinal SAS and reached the cranial CSF spaces within 2-h, which was interpreted to improve solute distribution to the brain compared to without a flush. To our knowledge, multiple flushes and flush timing has not been non-clinically or clinically investigated. However, repeated bolus injections have been shown to have a therapeutic effect in context of intrathecal pain and spasticity therapeutics: improved functional scores, lower 24-h opioid dose, and less dose escalation (McRoberts et al., 2017). Additionally, multiple bolus doses showed reduced potential for intrathecal fibrosis in dogs when compared to continuous infusion, though this may be accounted for by a drug specific effect and may not hold true for all drugs (Hildebrand et al., 2019). Repeated bolus injections, and therefore perhaps flushes, may also allow lower injection volumes and more rapid CSF pressure recovery post-injection.

### Increasing Lumbar Spine Injection Bolus Rate by ~2X Had Little Impact on Solute Transport to the Brain

Increasing lumbar spine bolus injection rate did not increase solute transport to the brain (Figure 5, Table 4). It has been hypothesized that a greater bolus injection rate may increase solute spread due to potential to increase turbulence at the needle tip that could improve diffusion and mixing of the injected solute (Eisenach et al., 2003; Buchser et al., 2004). Similarly, within intrathecal drug delivery, investigators have considered that increasing an acute bolus injection rate may increase solute transport to the brain. In the present study, we increased the bolus injection rate from 5 to 13.3 mL/min and did not observe any statistically significant differences between the rates. The difference in findings may be a result of differing flow regimes. Typically, intrathecal drug delivery has been used to treat chronic pain and, in those cases, pain medication is often administered chronically in slow doses over the course of several hours or even days on the order of 0.01–0.02 mL/min (Buchser et al., 2004; Flack and Bernards, 2010). This is a value 200–300X slower than that imposed in our acute dosing study that ranged from 5 to 13 mL/min, and thus may not be directly comparable for the scenarios analyzed.

### Use of a Lumbar Placed Catheter Resulted in Greater Solute Distribution to the Brain Compared to LP Needle

On average, the results for the lumbar puncture catheter experiments delivered approximately twice as much solute to the brain compared to the results of the lumbar puncture needle experiments (p < 0.0001). When the catheter was oriented inferior to superior with the equivalent protocol, a statistically significant (*p* = 0.026) increase of 1.3 %ID to the brain was quantified. We hypothesized that this is a result of cranial-directed flow velocities originating from the catheter tip during drug injection. To test, the catheter tip was oriented orthogonal to the neuroaxis. Thus, to test the potential impact of device orientation, an additional experiment was conducted with the LP catheter at the same location as the LP needle but oriented in the anterior to posterior direction. This experiment showed no significant difference between the LP needle and catheter. These findings agree with a computational study conducted by Pizzichelli et al. (Pizzichelli et al., 2017) that found catheter angle and position could impact drug penetration to the spinal cord.

### Early Pharmacokinetic Solute Transport in the *in vitro* CSF System Agrees With *in vivo* Human Studies

Research has indicated that subject-specific CSF flow can be a factor leading to changes in CSF solute transport within humans (Edeklev et al., 2019; Eide et al., 2021; Halvorsen et al., 2021; Ringstad and Eide, 2021). Thus, because the current study was formulated based on subject-specific CSF flow boundary conditions, we expect results to be representative *in vivo* flow phenomena, but not identical. A study by Verma et al. quantified CSF-brain molecular exchange, neuraxial spread, and CSF-peripheral clearance in 15 healthy human volunteers after intrathecal injection of artificial CSF and Technetium-99 DPTA and observed signal translocation within the cranial cisterns and the brain parenchyma by 3-h post-injection (Verma et al., 2020). Another study measured glymphatic flow in a single 55-year-old male and found traces of gadobutrol delivered intrathecally in the cisterna magna between 1- and 3-h post-injection (Watts et al., 2019). A study by Ringstad et al. observed contrast of gadobutrol in the foramen magna after 20-min and a later study by the group observed contrast in the cerebellum between 2- and 4-h post IT administration in eight healthy volunteers (Ringstad et al., 2017, 2018). In the present study, we observed solute transport within the cranial region between 1- and 3- h post lumbar intrathecal injection, indicating spatial-temporal agreement with these *in vivo* studies.

It should be noted that our model was constructed with a rigid material while the human spinal anatomy, in specific, the thecal sac, is deformable. As such, the *in vivo* CSF flow pulsation phase and amplitude vary along the neuroaxis with nearly zero flow pulsation at the spinal termination. Thus, we expect that our model results lack accuracy in predicting solute dispersion near the spinal termination, for example, in the case of spinal anesthetics that are aimed to remain near the lumbar spine. The primary focus of this study was to model spatial-temporal solute distribution to the brain. In this context, our spatial-temporal CSF solute distribution show similarity to several *in vivo* studies in the literature as described above. Additionally, our study parametrically investigated changes in %ID to the brain and neuraxial AUC distributions rather than baseline magnitude of drug concentrations. As such, we expect the parametric impact of the different injection parameters to be similar even with moderate changes in CSF flow dynamics and/or geometry. However, future research should be applied to understand exactly what degree thecal sac deformation can alter solute transport to the brain.

### Limitations

There are several *in vitro* modeling limitations that have been previously described by our group (Sass et al., 2017). A primary limitation of the study was that the subject-specific 3D model used in this study neglects to assess drug absorption into the tissues of the CNS. Our approach was therefore to quantify early solute transport within a short time scale post-injection. Depending on the solute absorption, the results can vary substantially and could be modeled in future work by integrating molecular dynamics of the solute and the CNS. All experiments were conducted at room temperature in a rigid model without physiological feedback, which would result in slightly different molecular diffusion and viscosity compared to a human body. In this respect, the addition of a CSF pressure monitoring system in a deforming model could be incorporated for future work. Furthermore, drug specific kinetics have been shown to play an important role in the rate of drug dispersion and tissue uptake (Tangen et al., 2017). This study consisted of a generalized model of solute transport using an aqueous solution of fluorescein to represent a small molecule drug. Additionally, studies have shown that the pulsation rate has a significant effect on solute transport (Hsu et al., 2012; Tangen et al., 2017). A single, consistent, and idealized waveform was used in this study for which to make parametric comparisons across injection scenarios. Previously published studies have shown that the effects of CSF amplitude and frequency oscillations are important factors in solute transport (Khani et al., 2022); however, the primary objective of this study was to investigate the parametric changes of injection protocols. This subject-specific model also utilized semi-idealized geometry that may not fully represent diseased cases, such as syringomyelia or Chiari malformation (Sass et al., 2017). Future studies should investigate solute transport within the CSF for diseased cases. Further, the introduction of a cranio-cervical pulsation, specifically within the highly vascularized cerebellar area would allow for further resemblance to *in vivo* (Watts et al., 2019; Khani et al., 2020a). Longer duration *in vitro* studies could be conducted to further investigate the agreement between trends observed *in vitro* and *in vivo*; *in vivo* studies have tracked drug dispersion up to 80-h (Watts et al., 2019) and *in vitro* studies up to 24-h (Khani et al., 2020a). Lastly, subject-specific *in vitro* vs. *in vivo* CSF transport has not been fully validated in this study.

## Conclusions

We addressed ITDD injection parameters within a complete CSF system of a 3D subject-specific human model. These parameters included: bolus injection volume and rate, flush volume and rate, injection location, type of device, and flush timing. For all simulations analyzed, the overall efficiency of solute delivery to the brain was limited with <12% of ID reaching the intracranial space. Because delivery efficiency to the brain was low, even small improvements in %ID to the brain due to injection protocols were found to potentially have substantial impact, in particular, when combined together. Within that context, flush volume had the most significant impact on increasing solute transport to the brain within 3-h (+3.9%ID, *p* = 0.009). This was followed by flush rate (+1.6%ID, *p* = 0.038), type of device (+1.5%ID, *p* = 0.026). Bolus injection volume (+1.3%ID), injection location (+1.2%ID), flush timing (+1.2%ID), and bolus injection rate (−0.6%ID) did not show a statistically significant impact. In combination, these findings indicate that lumbar spine ITDD injection protocols can be optimized by modest changes in injection parameters and devices to improve therapeutic delivery of drugs to the brain.

## Data Availability Statement

The original contributions presented in the study are included in the article/Supplementary Material, further inquiries can be directed to the corresponding author.

## Author Contributions

BM: study conception and design. AS: acquisition of data. AS, GB, BM, and DS: analysis and interpretation of data and critical revision. AS and BM: drafting of manuscript. All authors contributed to the article and approved the submitted version.

## Funding

This work was supported by Genentech Inc., an Institutional Development Award (IDeA) from the National Institute of General Medical Sciences (NIGMS) of the National Institutes of health (NIH) under Grant #P20GM1033408 and #4U54GM104944-04TBD, National Institute of Neurological Disorders and Stroke Grant# R01NS111283, and University of Idaho Vandal Ideas Project.

## Conflict of Interest

BM is an employee at Alcyone Therapeutics. BM has received research funding from Biogen Inc., Genentech Inc., Voyager Therapeutics, KBR Wyle, Alcyone Lifesciences Inc., Minnetronix Inc., and Voyager Therapeutics. BM has served as a consultant to Flux Neuroscience, Genentech, Roche, Minnetronix, SwanBio Therapeutics, Praxis Medicines, Cerebral Therapeutics, CereVasc, InviCRO, Neurosyntec, Behavior Imaging, Anuncia Medical, and Voyager Therapeutics. BM has been a scientific advisory board member for Alcyone Lifesciences, Anuncia Medical, Chiari and Syringomyelia Foundation, The International Society for Hydrocephalus and CSF Disorders, and The International CSF Dynamics Society. DS has received research funding from Genentech Inc. MB and JH were employed by Genentech, Inc., A member of the Roche Group. The remaining authors declare that the research was conducted in the absence of any commercial or financial relationships that could be construed as a potential conflictof interest.

## Publisher's Note

All claims expressed in this article are solely those of the authors and do not necessarily represent those of their affiliated organizations, or those of the publisher, the editors and the reviewers. Any product that may be evaluated in this article, or claim that may be made by its manufacturer, is not guaranteed or endorsed by the publisher.

## Figures and Tables

**Table:** 

Cmax	Maximum concentration
N1, n2	Sample size for group 1 and 2
Xbar	Population mean
Xbar	Pooled standard deviation

## Supplementary Material

Supplementary Figure 1 Effect of flush volume by average AUC and spatial temporal distribution comparison. There is a significant difference observed between the AUC trends, as the protocol with the higher flush volume, 3HUM3, delivered more to the brain than equivalent protocol with the lower flush volume. This can also be observed in the spatial temporal trends. Immediately after injection, 3HUM3 had a steeper slope of solute distribution, thereby pushing more of the solute cranially and reaching a location of ~15 cm. The solute distribution did not reach the same location, −15 cm, until ~30 min after the injection.

Supplementary Figure 2 Effect of flush rate by average AUC and spatial temporal distribution comparison. There is no significant difference between the AUC trends and the spatial temporal distribution plots; however, it can be observed that within the brain region (0–10 cm) on the AUC plot, the case with the higher flush rate, 1HUM3, delivered slightly more solute to the brain.

Supplementary Figure 3 Effect of device by average AUC and spatial temporal distribution comparison. There is a noticeable difference between the plots. Within the AUC trends, a significant difference can be observed in the spinal region (0 to −60 cm), however, the differences decrease in the brain region (0–10 cm). The spatial temporal distribution also indicates a difference in spread near the spinal region and similar distribution near the brain region.

Supplementary Figure 4 Effect of bolus injection volume by average AUC and spatial temporal distribution comparison. Similar to Supplementary Figure 1, significant differences can be observed across both the AUC trends and the spatial temporal distribution. The AUC trends indicate a significant difference across all regions, with the case containing the higher bolus injection volume delivering more solute cranially toward the brain. Also, similar to Supplementary Figure 1, the case with the large bolus injection volume displays a more rapid distribution of solute immediately after injection, with the smaller bolus injection volume producing a slower distribution rate.

Supplementary Figure 5 Effect of injection location by average AUC and spatial temporal distribution comparison. The case with the higher injection location, 4HUM2, displayed less solute transport within the lumbar region (−50 to −60 cm), which agrees with the higher injection location, and is also observed in the 4HUM2 spatial temporal distribution plot.

Supplementary Figure 6 Effect of bolus rate by average AUC and spatial temporal distribution comparison. Despite a higher bolus rate, no noticeable differences were observed between 2HUM1 and 2HUM2.

Supplementary Figure 7 Effect of delayed flush by average AUC and spatial temporal distribution comparison. The decrease in solute concentration after injection in 5HUM1 is believed to be a result of dilution caused by the large flush volume. While the spatial temporal trends are difficult to compare, the AUC trends indicate little difference in the brain region.

Supplementary Figure 8 Boxplots of the mean lumbar puncture needle and catheter experiments. On average, protocols conducted with a lumbar puncture catheter (3HUM1–4HUM2) showed nearly 2X solute transport to the brain when compared to lumbar puncture needle protocols (1HUM1–2HUM2).

Supplementary Video 1 Average AUC over time for all cases. There is a strong initial difference between cases, however, these differences decrease over time.

## Abbreviations

AUC: Area Under the Curve
BBB: Blood Brain Barrier
CI: Confidence Interval
CNS: Central Nervous System
CSF: Cerebrospinal Fluid
DR: Dynamic Range
ID: Injected Dose
IT: Intrathecal
ITDD: Intrathecal Drug Delivery
MRI: Magnetic Resonance Imaging
SAS: Subarachnoid Space.
