# Supplementary material for: Investigation of Human Intrathecal Solute Transport Dynamics Using a Novel in vitro Cerebrospinal Fluid System Analog
Source: Front Neuroimaging. 2022 Jun 23;1:879098. doi: 10.3389/fnimg.2022.879098 (PMC10406265; doi:10.3389/fnimg.2022.879098)
Supplement: Supplementary Figure 1 — Effect of flush volume by average AUC and spatial temporal distribution comparison. There is a significant difference observed between the AUC trends, as the protocol with the higher flush volume, 3HUM3, delivered more to the brain than equivalent protocol with the lower flush volume. This can also be observed in the spatial temporal trends. Immediately after injection, 3HUM3 had a steeper slope of solute distribution, thereby pushing more of the solute cranially and reaching a location of ~15 cm. The solute distribution did not reach the same location, −15 cm, until ~30 min after the injection. [file Data_Sheet_1.docx]

**Supplementary Figures**

**Supplementary Figure 1.** Effect of flush volume by average AUC and spatial temporal distribution comparison. There is a significant difference observed between the AUC trends, as the protocol with the higher flush volume, 3HUM3, delivered more to the brain than equivalent protocol with the lower flush volume. This can also be observed in the spatial temporal trends. Immediately after injection, 3HUM3 had a steeper slope of solute distribution, thereby pushing more of the solute cranially and reaching a location of approximately -15 cm. The solute distribution did not reach the same location, -15 cm, until approximately 30 minutes after the injection.

**Supplementary Figure 2.** Effect of flush rate by average AUC and spatial temporal distribution comparison. There is no significant difference between the AUC trends and the spatial temporal distribution plots; however, it can be observed that within the brain region (0 to 10 cm) on the AUC plot, the case with the higher flush rate, 1HUM3, delivered slightly more solute to the brain.

**Supplementary Figure 3.** Effect of device by average AUC and spatial temporal distribution comparison. There is a noticeable difference between the plots. Within the AUC trends, a significant difference can be observed in the spinal region (0 to -60 cm), however, the differences decrease in the brain region (0 to 10 cm). The spatial temporal distribution also indicates a difference in spread near the spinal region and similar distribution near the brain region.

**Supplementary Figure 4.** Effect of bolus injection volume by average AUC and spatial temporal distribution comparison. Similar to Supplementary Figure 1, significant differences can be observed across both the AUC trends and the spatial temporal distribution. The AUC trends indicate a significant difference across all regions, with the case containing the higher bolus injection volume delivering more solute cranially towards the brain. Also, similar to Supplementary Figure 1, the case with the large bolus injection volume displays a more rapid distribution of solute immediately after injection, with the smaller bolus injection volume producing a slower distribution rate.

**Supplementary Figure 5.** Effect of injection location by average AUC and spatial temporal distribution comparison. The case with the higher injection location, 4HUM2, displayed less solute transport within the lumbar region (-50 to -60 cm), which agrees with the higher injection location, and is also observed in the 4HUM2 spatial temporal distribution plot.

**Supplementary Figure 6.** Effect of bolus rate by average AUC and spatial temporal distribution comparison. Despite a higher bolus rate, no noticeable differences were observed between 2HUM1 and 2HUM2.

**Supplementary Figure 7.** Effect of delayed flush by average AUC and spatial temporal distribution comparison. The decrease in solute concentration after injection in 5HUM1 is believed to be a result of dilution caused by the large flush volume. While the spatial temporal trends are difficult to compare, the AUC trends indicate little difference in the brain region.

**Supplementary Figure 8.** Boxplots of the mean lumbar puncture needle and catheter experiments. On average, protocols conducted with a lumbar puncture catheter (3HUM1 – 4HUM2) showed nearly 2X solute transport to the brain when compared to lumbar puncture needle protocols (1HUM1 – 2HUM2).

**Supplementary Figure 1**


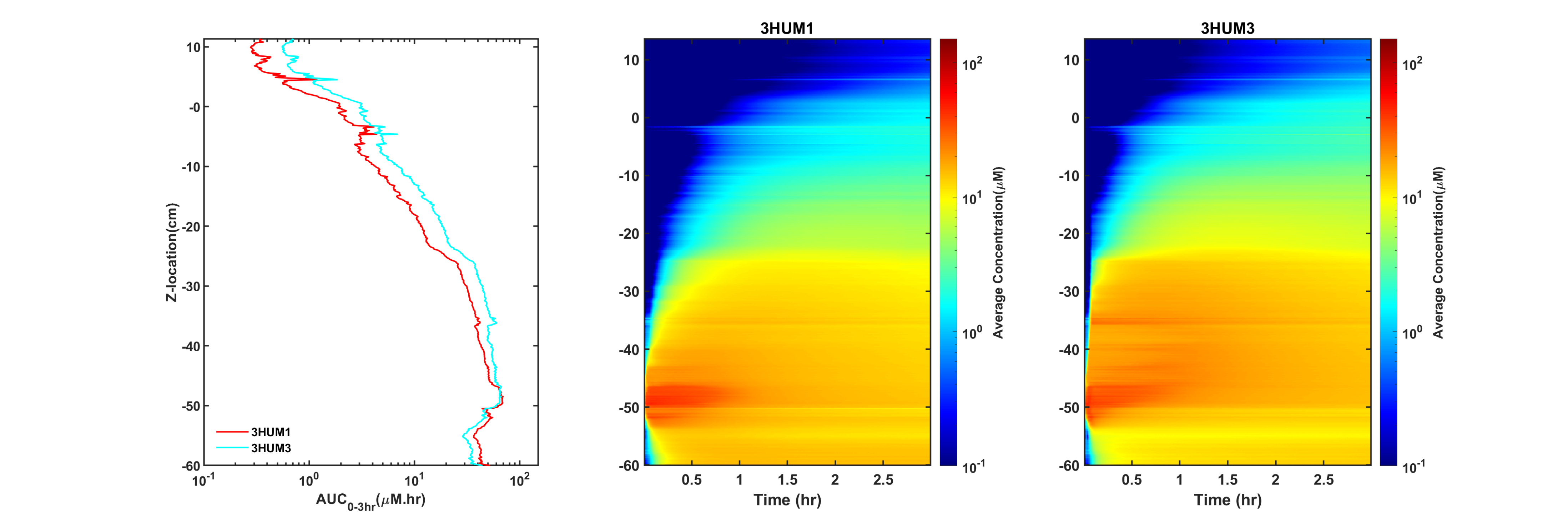

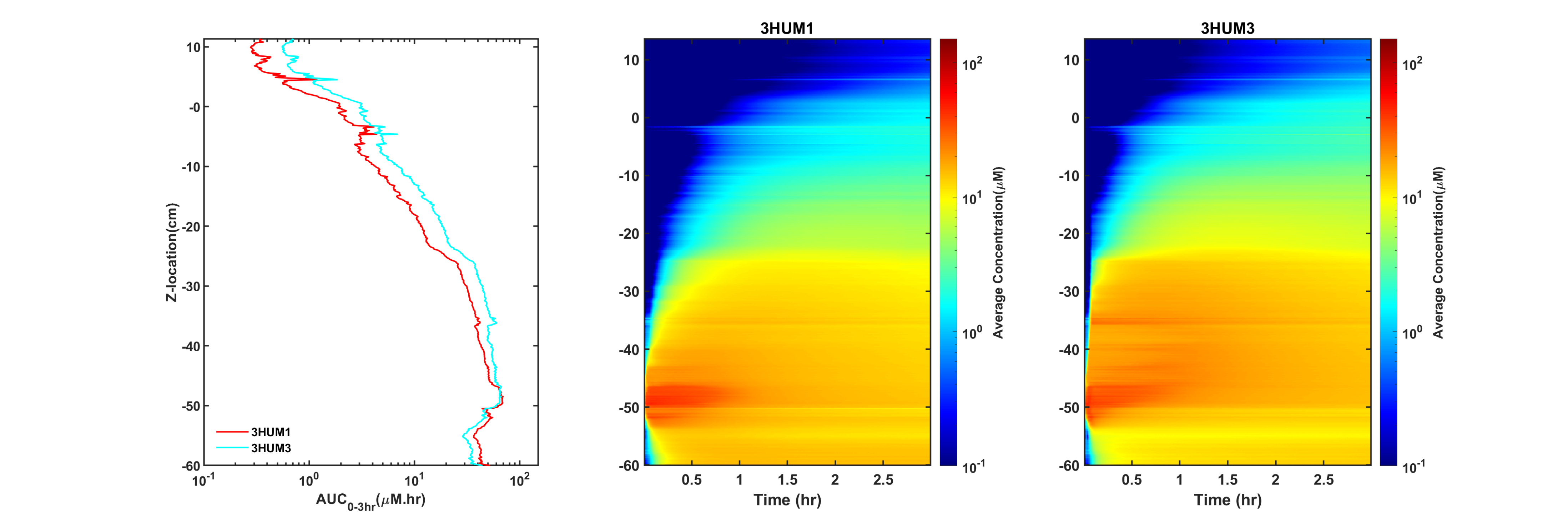

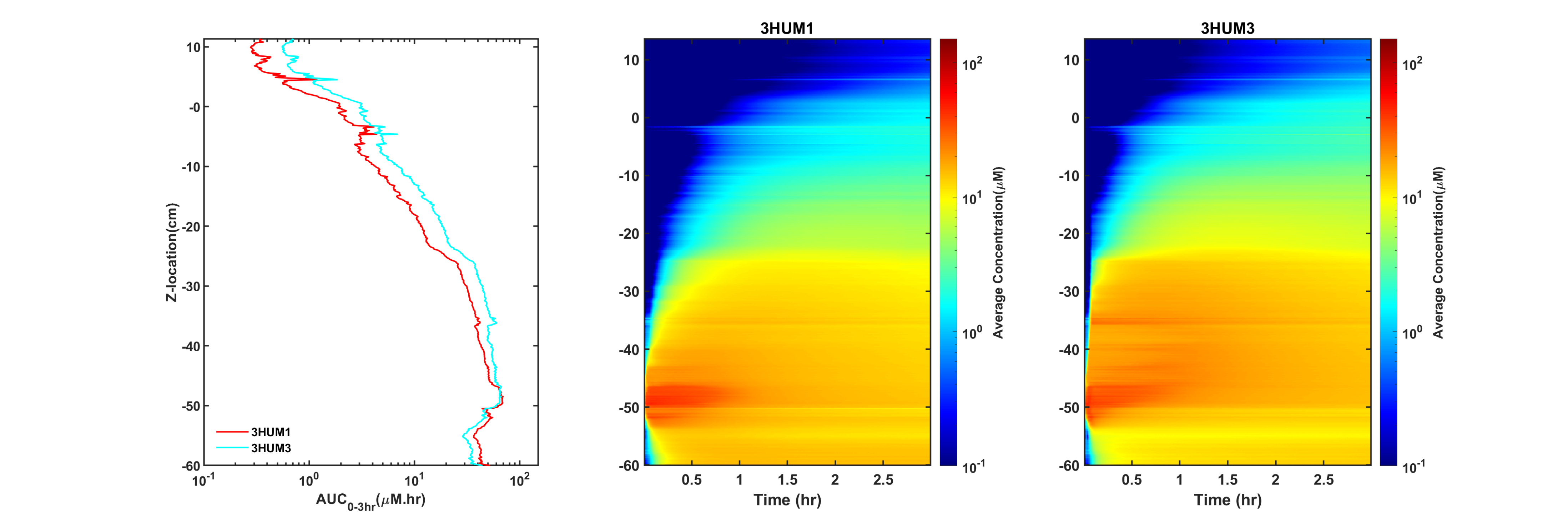


**Supplementary Figure 2**


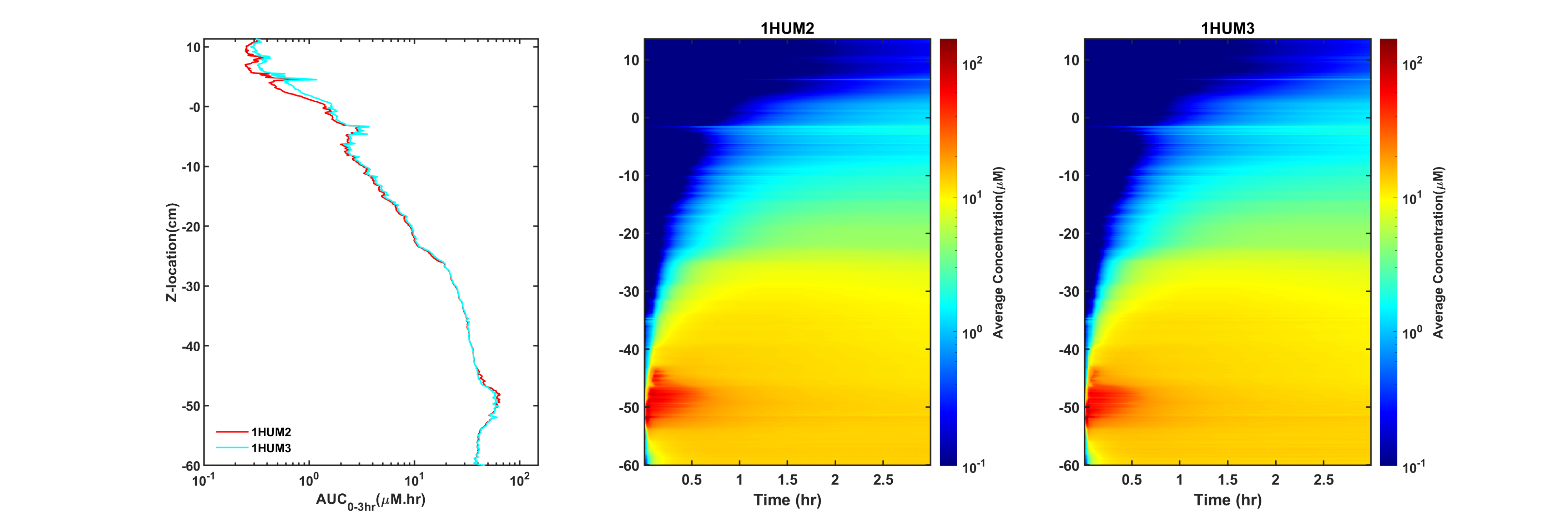

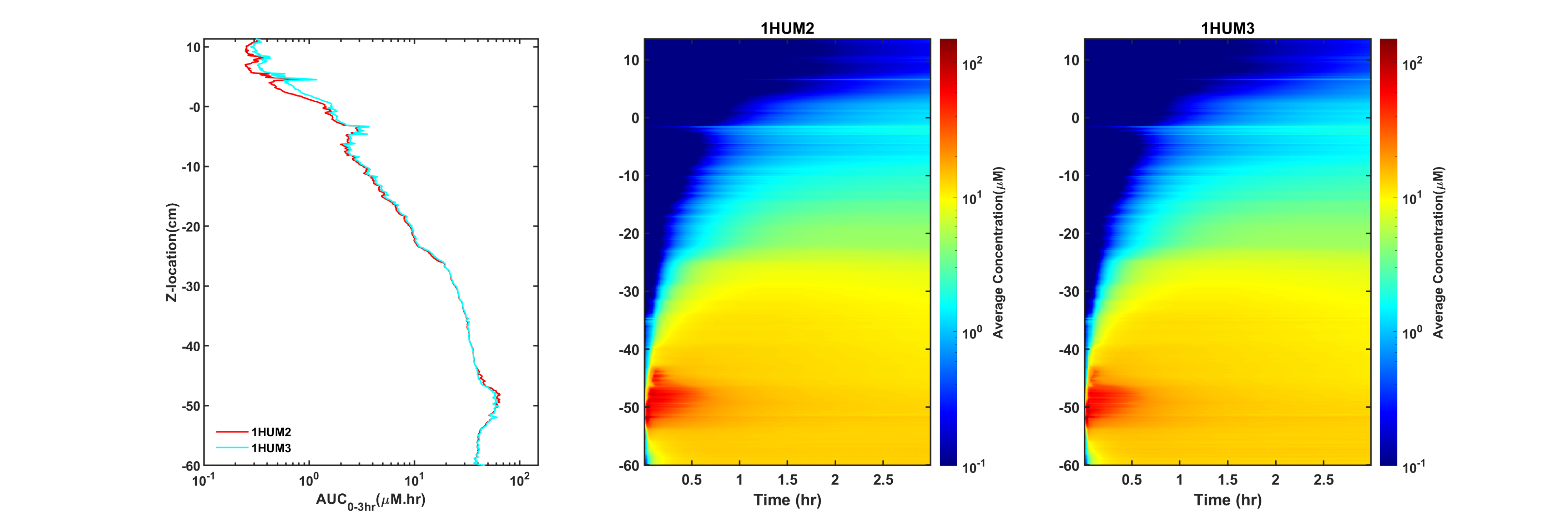


**Supplementary Figure 3**


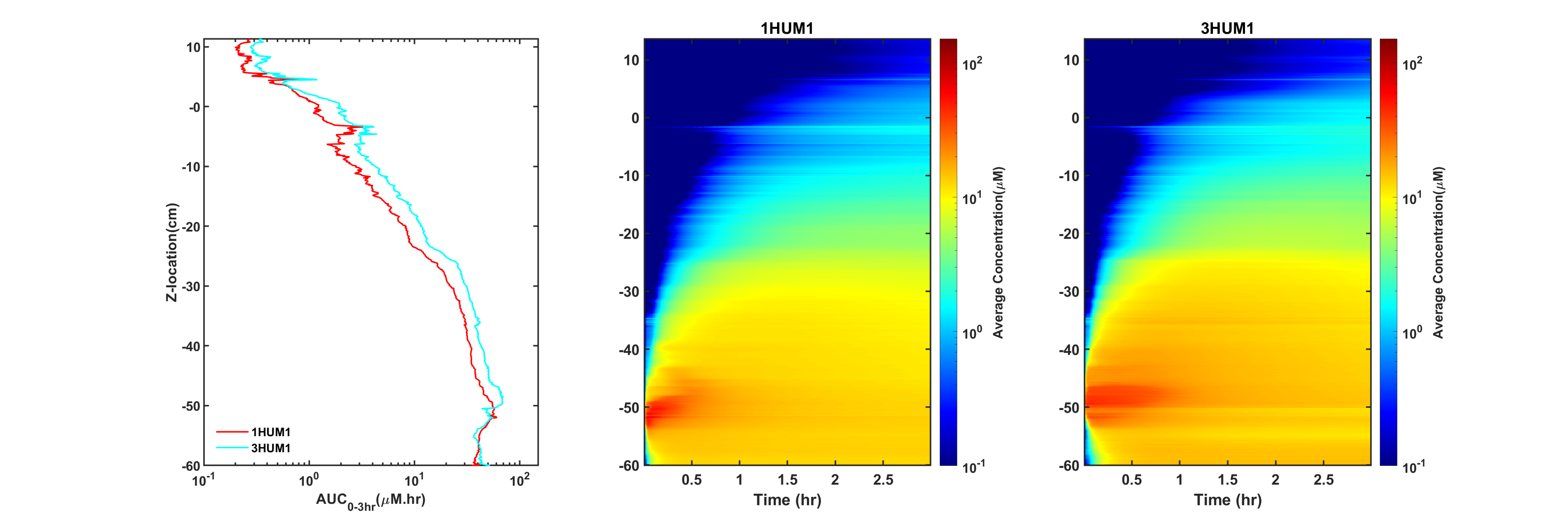

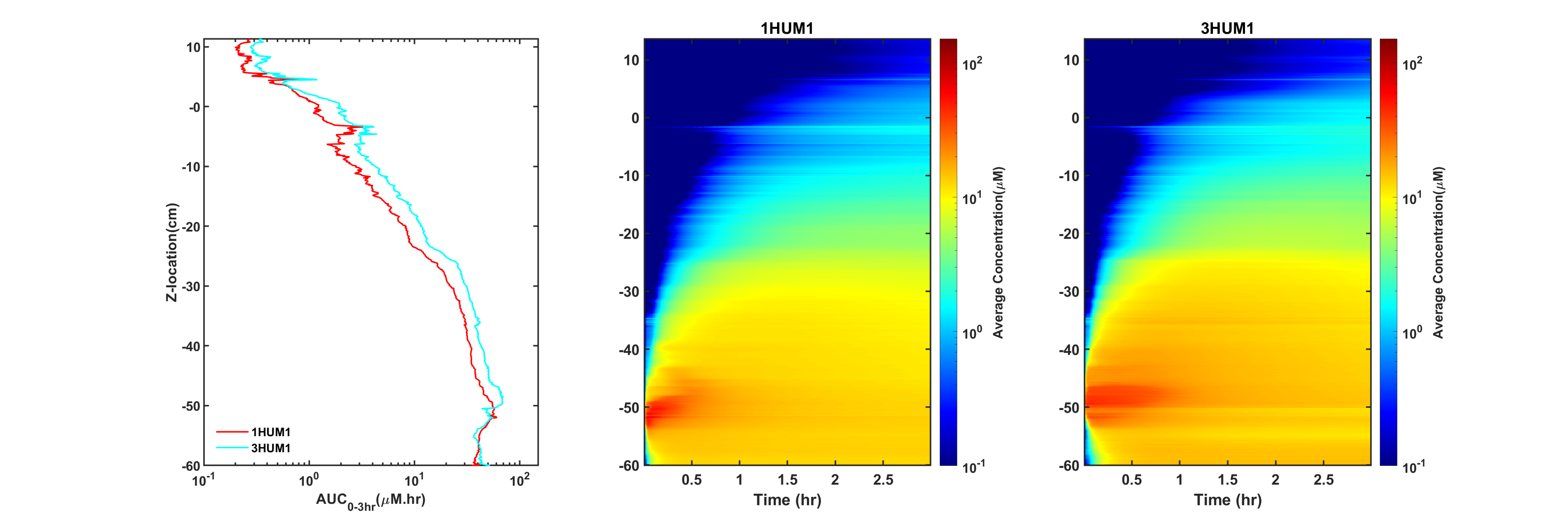


**Supplementary Figure 4**


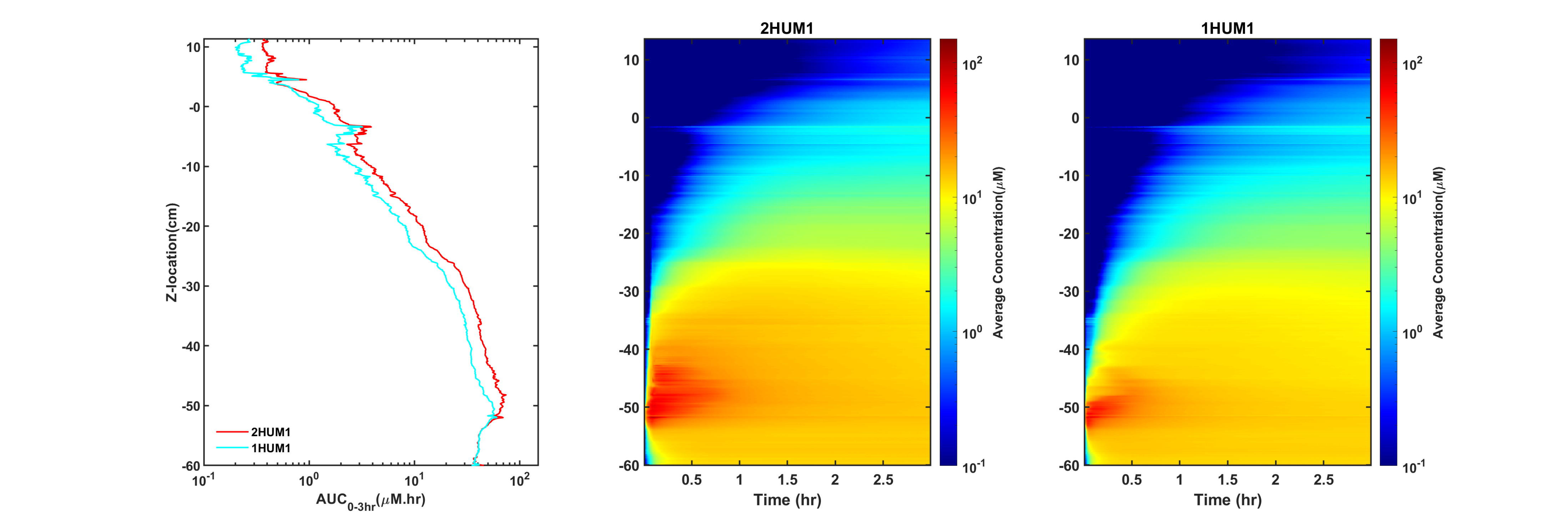

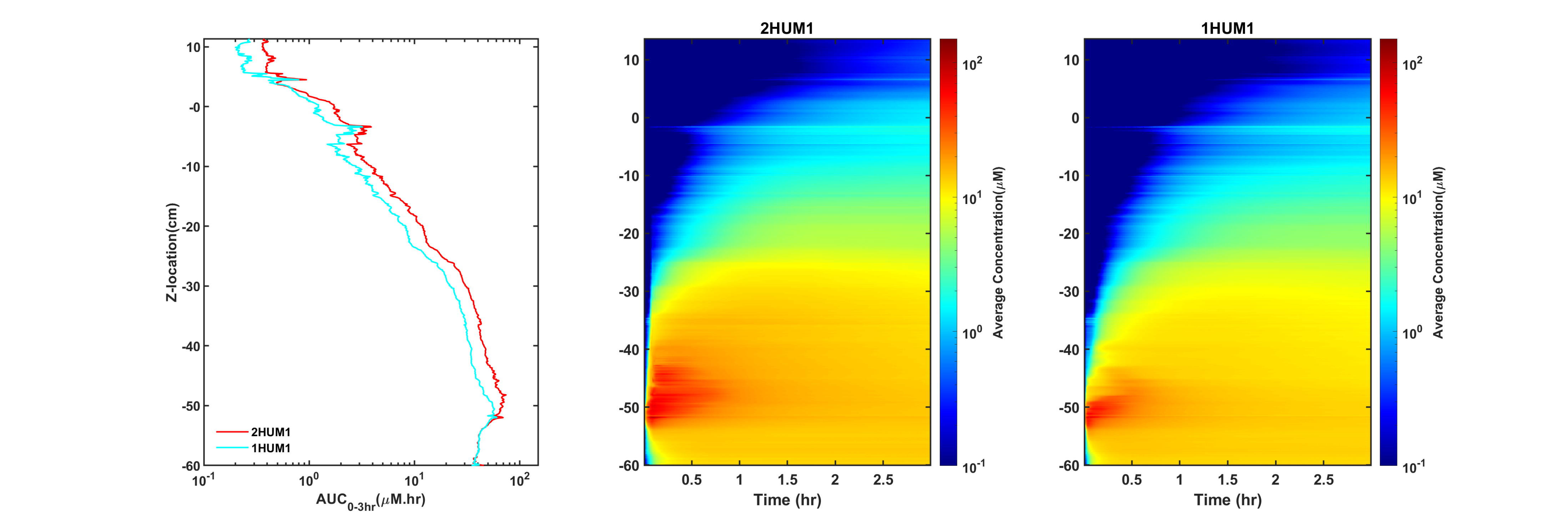


**Supplementary Figure 5**


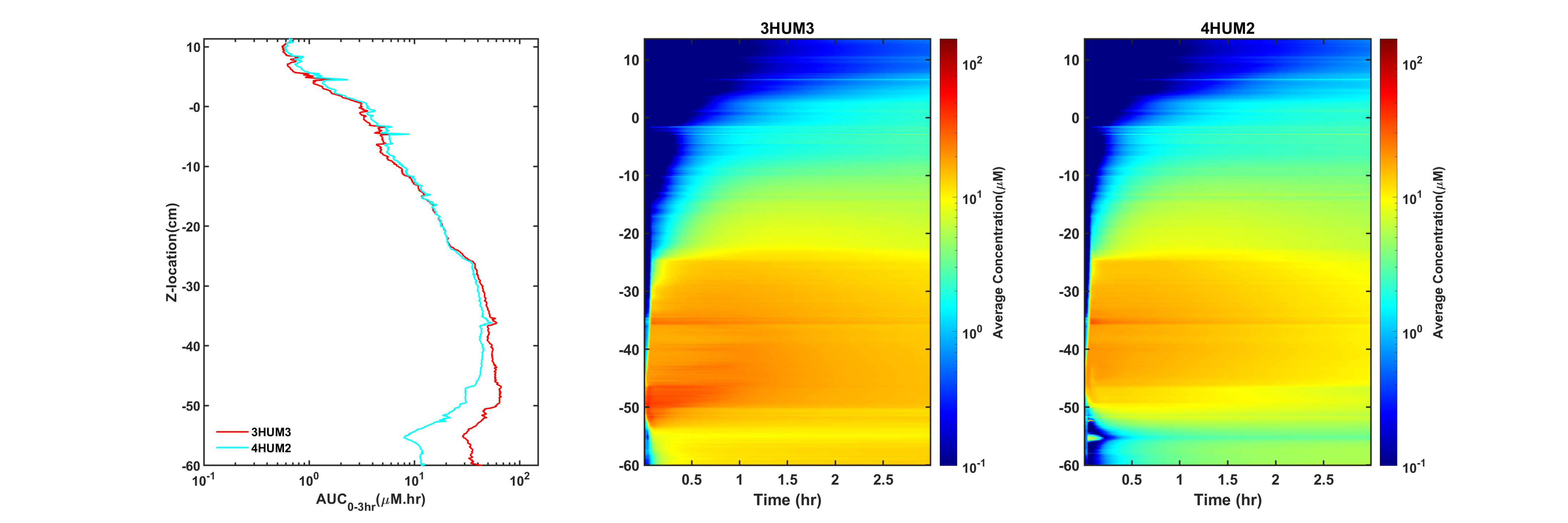

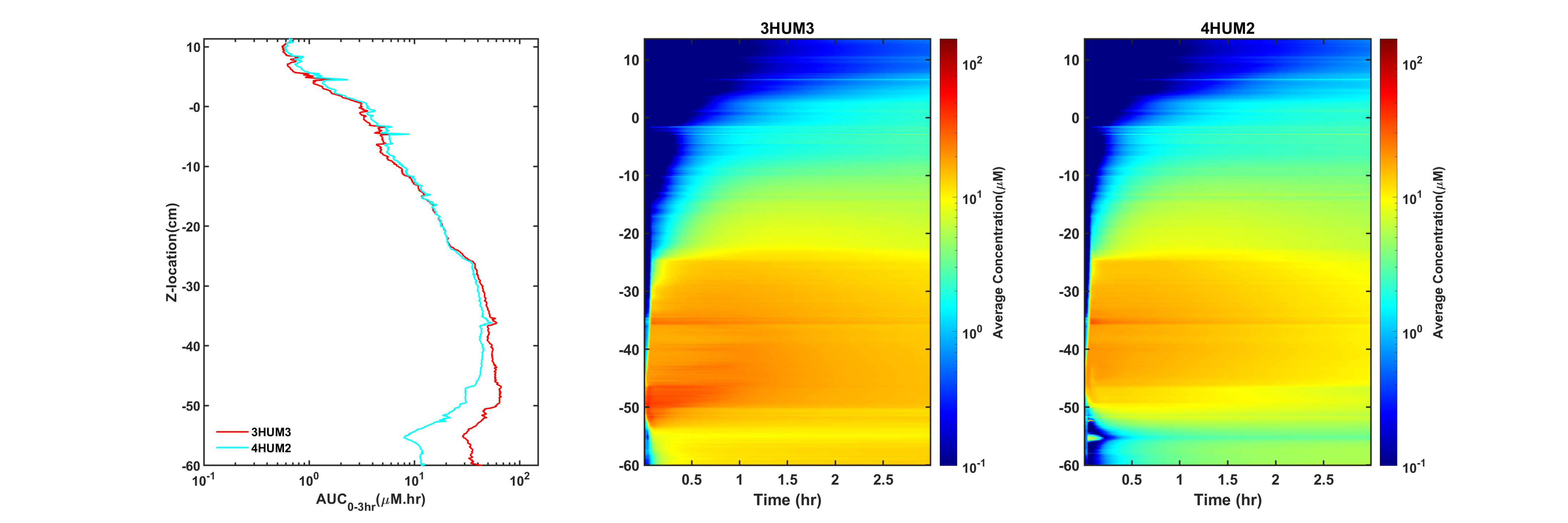


**Supplementary Figure 6**


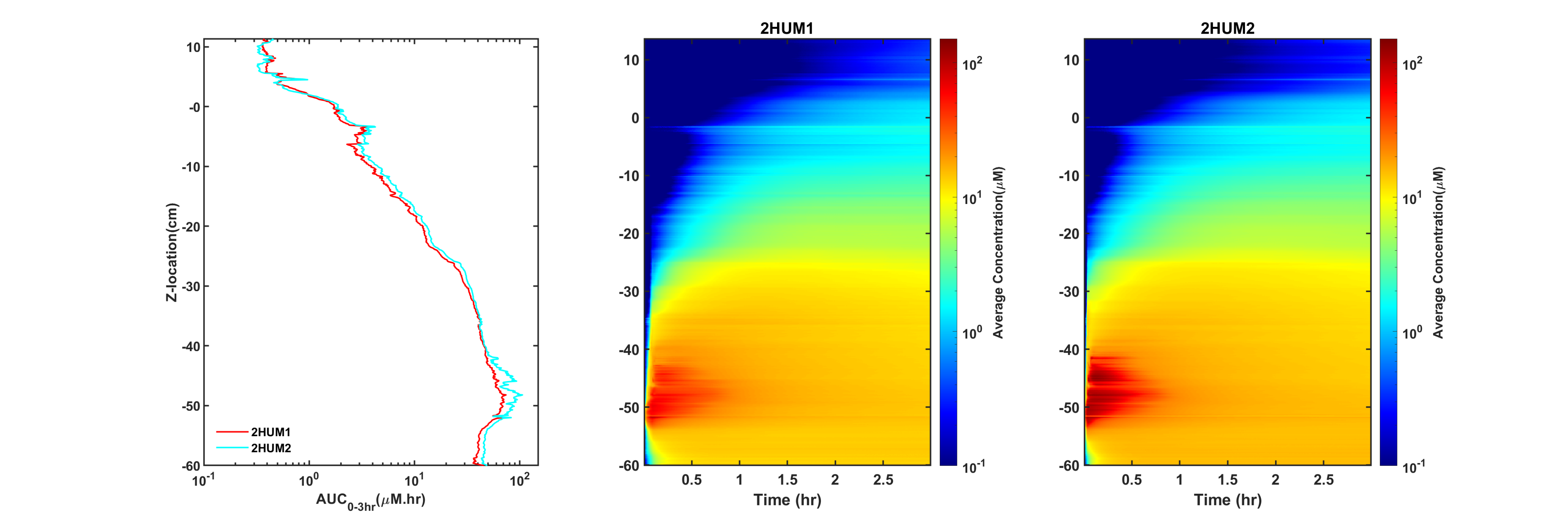

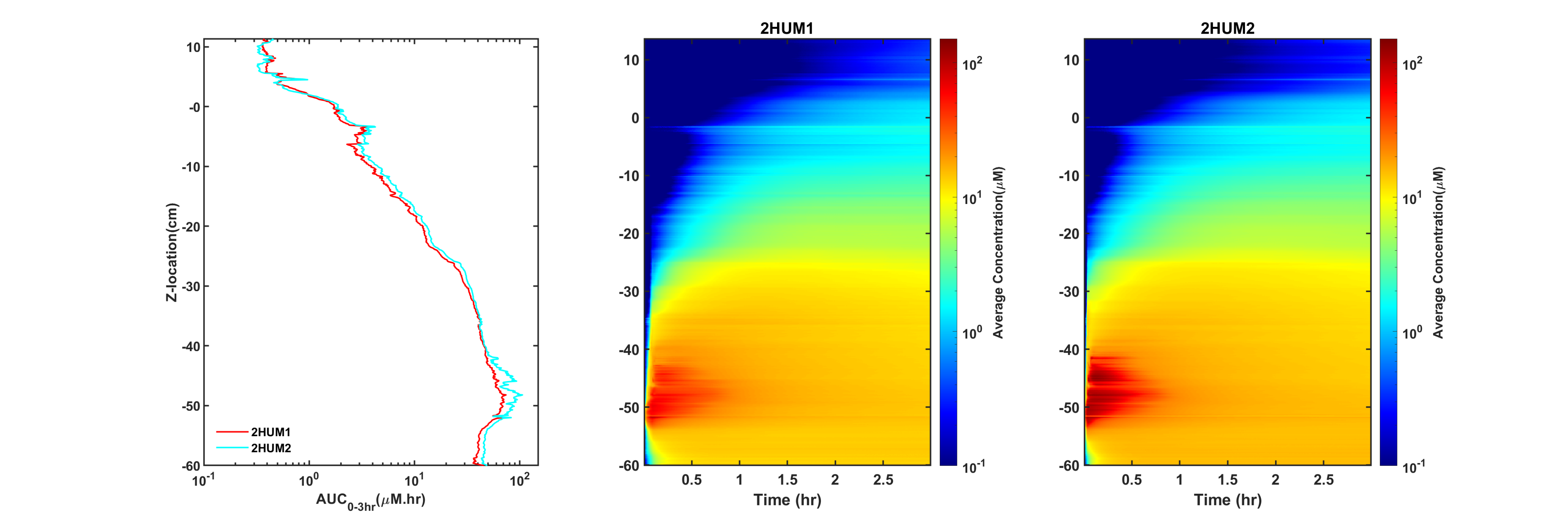


**Supplementary Figure 7**


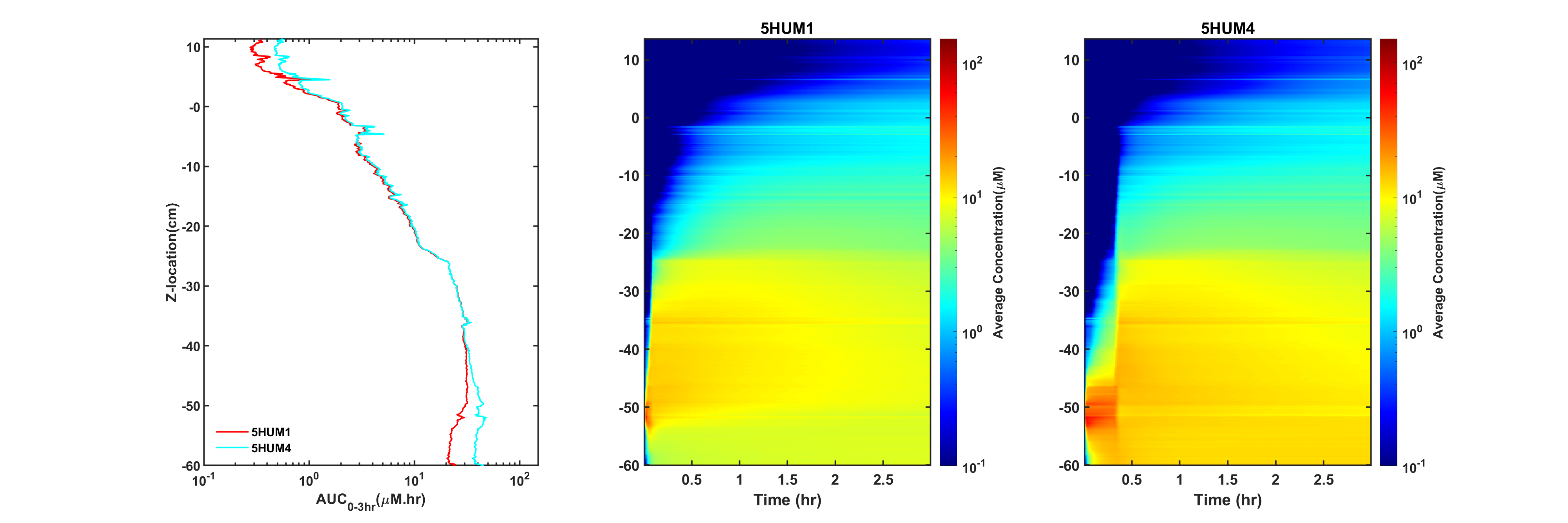

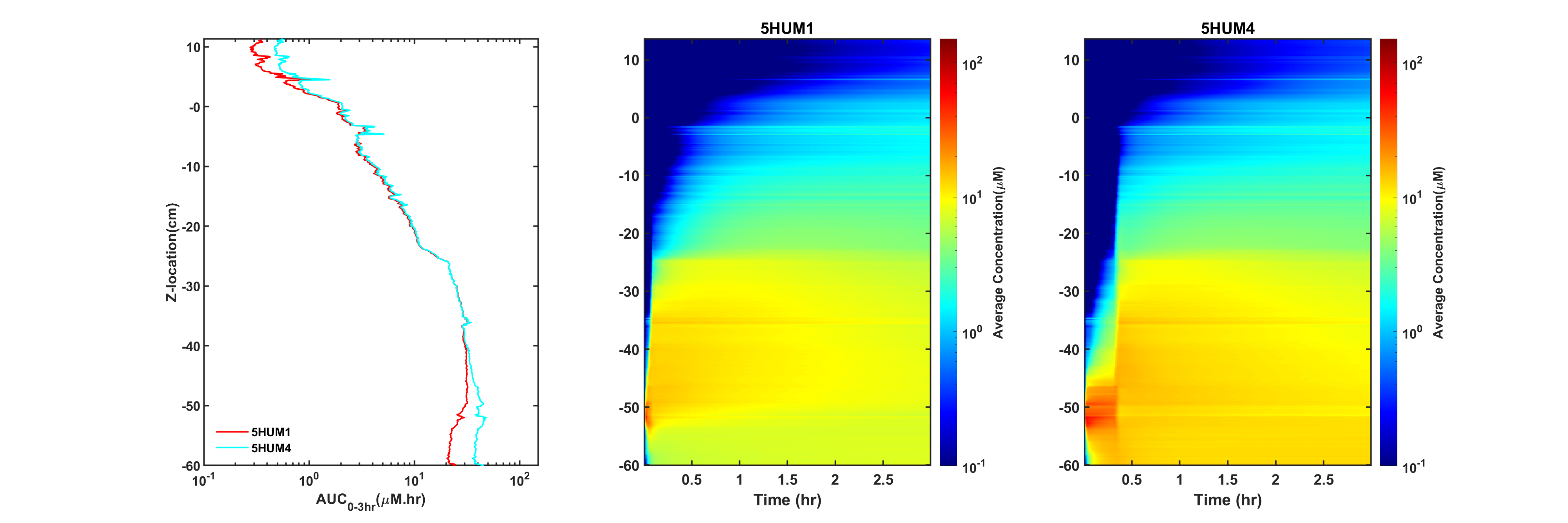


**Supplementary Figure 8**

**
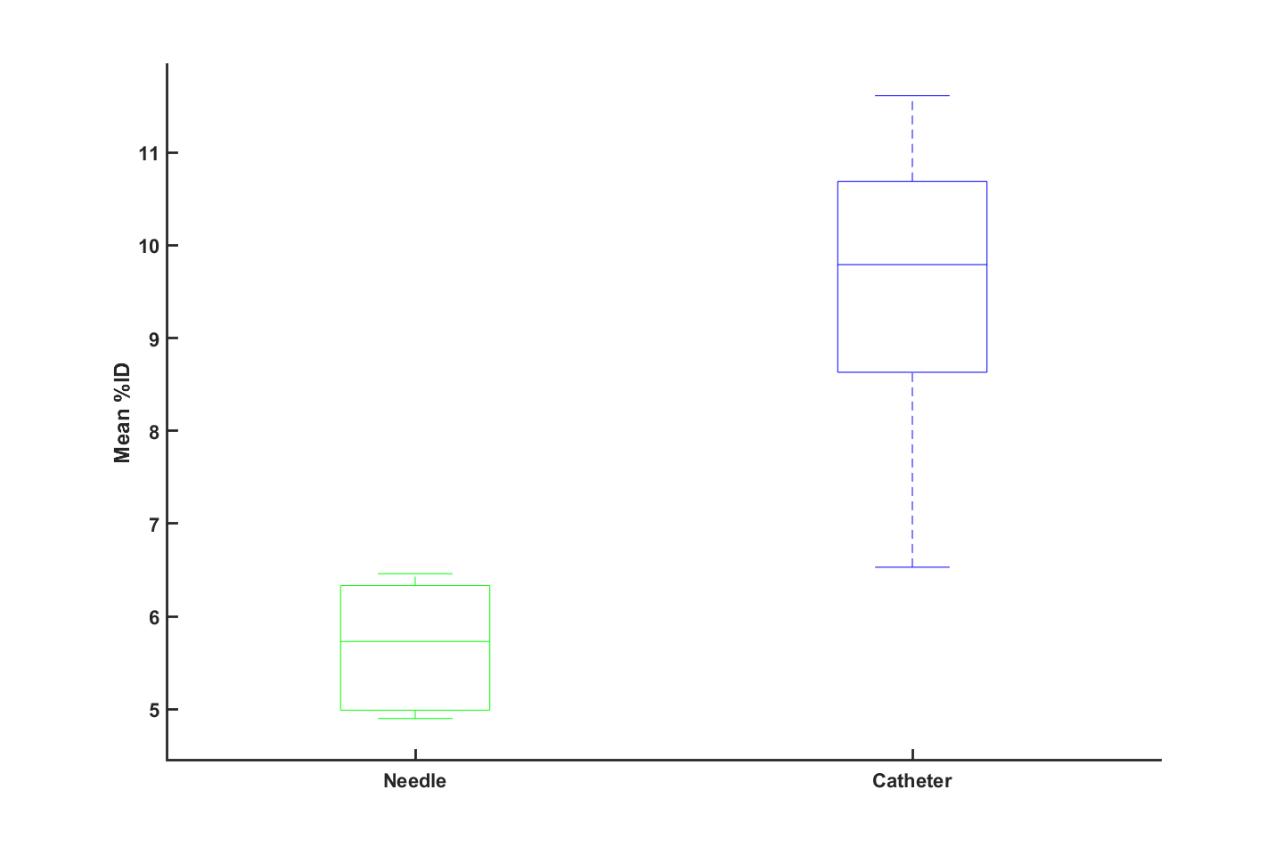
**
